# A systematic review of treatment strategies to combat acute and chronic rejection episodes in vascularized composite allotransplantation

**DOI:** 10.3389/fimmu.2026.1733221

**Published:** 2026-02-02

**Authors:** Leonard Knoedler, Tobias Niederegger, Thomas Schaschinger, Gabriel Hundeshagen, Robert Munzinger, Max Heiland, Curtis L. Cetrulo, Alexandre G. Lellouch

**Affiliations:** 1 Charité–Universitätsmedizin Berlin, corporate member of Freie Universität Berlin and Humboldt-Universität zu Berlin, Department of Oral and Maxillofacial Surgery, Berlin, Germany; 2 Division of Plastic and Reconstructive Surgery, Cedars-Sinai Medical Center, Los Angeles, CA, United States; 3 University of Heidelberg, Medical Faculty Heidelberg, Heidelberg, Germany; 4 Department of Hand, Plastic and Reconstructive Surgery, Burn Center, BG Trauma Hospital Ludwigshafen, Ludwigshafen, Germany; 5 Department of Plastic and Hand Surgery, University of Heidelberg, Ludwigshafen, Germany; 6 Vascularized Composite Allotransplantation Laboratory, Massachusetts General Hospital, Harvard Medical School, Boston, MA, United States; 7 Innovative Therapies in Haemostasis, INSERM UMR-S 1140, University of Paris, Paris, France

**Keywords:** allotransplant, rejection, rejection treatment, vascularized composite allotransplantation, VCA

## Abstract

**Background:**

Vascularized composite allotransplantation (VCA) offers unique reconstructive solutions for severe tissue loss, restoring form and function. Acute and chronic rejection remains a significant barrier, with acute episodes occurring in most recipients and chronic rejection persisting as the leading cause of graft failure. Unlike solid organ transplantation, VCA involves highly immunogenic tissues, like skin and mucosa, making rejection more frequent and challenging to manage.

**Methods:**

A systematic review was conducted following PRISMA 2020, searching PubMed/MEDLINE, EMBASE, and Web of Science for original human VCA studies reporting immunosuppressive protocols and outcomes in acute or chronic rejection. Quality was assessed using the Newcastle–Ottawa Scale and Level of Evidence; extracted data included demographics, regimens, rejection episodes, treatments, and graft survival.

**Results:**

Fourty-six studies (136 recipients) met inclusion criteria: upper extremity (n=69; 51%), face (n=33; 24%), abdominal wall (n=33; 24%), scalp and penile (each n=1; 0.7%). Acute rejection occurred in 81/136 (60%) within year 1, most often at POW 1–2 (n=52), 5–12 (n=42), and 13–52 (n=30). Severity was Banff grade I (n=49; 36%), II (n=73; 54%), III (n=50; 37%), and severe IV (n=1; 0.7%). Common symptoms included skin lesions (n=43; 32%), edema (n=32; 24%), erythema (n=29; 21%), and rash (n=15; 11%), with some experiencing numbness (n=4; 2.9%), tingling (n=5; 3.7%), or burning sensations (n=5; 3.7%). Corticosteroids were the mainstay (n=98; 72%)—methylprednisolone (n=31; 23%), clobetasol (n=15; 11%), and prednisone (n=11; 8.1%); tacrolimus was used in 49 (36%), including topical in 29 (21%). Other immunosuppressants included antithymocyte globulin (n=19; 14%), alemtuzumab (n=11; 8.1%), mycophenolate mofetil (n=11; 8.1%), and rituximab (n=6; 4.4%); basiliximab (n=4; 2.9%), sirolimus (n=2; 1.5%), and plasmapheresis (n=4; 2.9%) were used selectively. Monotherapy was used in 42 episodes, and dual therapy in 51, most commonly methylprednisolone plus topical tacrolimus (n=26).

**Conclusion:**

This review underscores the ongoing challenge of rejection in VCA and the need for improved treatment strategies, with corticosteroids, calcineurin inhibitors, and mycophenolate mofetil remaining standard while emerging biologicals offer promise. Acute rejection is often manageable yet threatens graft survival, whereas chronic rejection is less reported, likely under-recognized and harder to treat, underscoring need for novel immunomodulators, standardized protocols, and prevention to improve outcomes.

## Graphical Abstract

**Figure:**
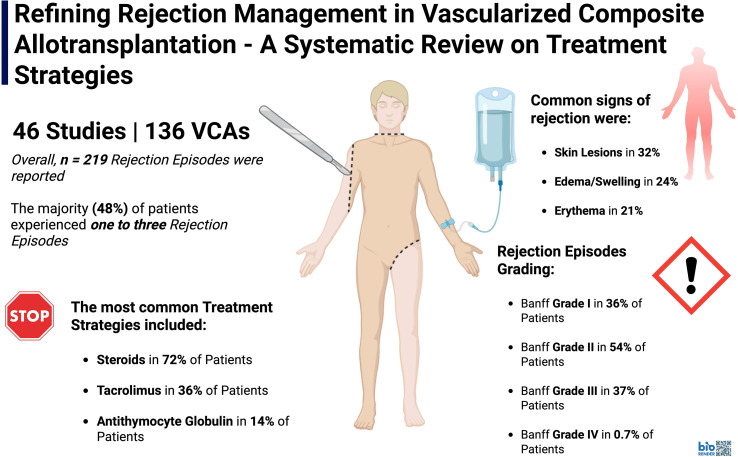

## 1 Introduction

Vascularized composite allotransplantation (VCA) is a life-changing procedure that offers potential restoration of lost function and appearance for patients with severe tissue defects. However, rejection remains a major problem, limiting the long-term success and widespread use of VCA. Unlike solid organ transplants (SOT), VCA includes multiple tissue types such as skin, mucosa, muscle, and nerves, making rejection more frequent and difficult to control (1–5).

Among all VCA types, face transplants experience the highest rejection rates, possibly due to the high proportion and immunogenicity of skin and mucosal tissue (6, 7). Acute rejection is most commonly observed within the first year post-transplant, with over 85% of recipients experiencing at least one episode, often within the first 3 to 6 months, though some early events may occur within 30 days. Chronic rejection, on the other hand, tends to develop after the first year, manifesting as progressive vasculopathy, fibrosis, or functional decline of the graft over time (8–10). Overall, chronic rejection is more rare and considered the leading cause of long-term graft failure, with 10-20% of face and upper extremity VCA recipients experiencing chronic rejection (2, 11, 12). Therefore, chronic rejection has been identified as the leading cause of graft loss and retransplantation (13–15). In summary, the high risk of rejection poses a significant barrier that hinders widespread clinical adoption of VCA and varies in certain types of VCA (16, 17).

Treating rejection in VCA depends on the severity and type of rejection. Mild acute rejection is usually managed with increased doses of corticosteroids, while more severe cases may require additional immunosuppressive drugs like tacrolimus (TAC) or mycophenolate mofetil (MMF) (10, 18). In cases of chronic rejection, effective treatment options remain limited. Chronic rejection in VCA lacks an established treatment and is often diagnosed alongside graft deterioration. Therapies like intravenous immunoglobulin (IVIG), plasmapheresis, and conversion to sirolimus have shown limited success, underlining the need for novel therapies and additional research to fill this gap in the literature (1, 19).

Overall, treating rejection in VCA remains challenging, limiting the widespread applicability of VCA surgery. Therefore, consolidation of existing literature is necessary to identify knowledge gaps. This could provide helpful insights for both VCA providers and patients and pave the way for further research. To fill this gap, this systematic review aims to explore current and emerging treatment strategies for rejection in VCA recipients.

## 2 Methods

This systematic review was conducted following the Preferred Reporting Items for Systematic Reviews and Meta-Analyses (PRISMA) 2020 guidelines. Given the heterogeneity in study designs, patient cohorts, and outcome measures, a narrative synthesis was chosen. The full study protocol is accessible at PROSPERO (CRD420251027621).

### 2.1 Systematic search

A comprehensive literature search was conducted across PubMed/MEDLINE, EMBASE, and Web of Science databases, covering studies published up to November 30, 2024, that focused on rejection treatment in vascularized composite allotransplantation (VCA) recipients. The search strategy combined two key components using the Boolean operator “AND” to refine the selection process. The first component included VCA-related terms, such as “vascularized composite allotransplantation”. The second component targeted rejection and immunosuppression-related terms, including “acute rejection”, “chronic rejection”, and “Banff classification”. MeSH-Terms as well as synonyms of each were applied accordingly. To ensure a comprehensive overview, cross-referencing of fitting studies was performed. The full search strategy is provided in the **Supplementary Table**. Studies were eligible for inclusion if they provided original data on treatment strategies for acute or chronic rejection in human VCA recipients, covering interventions such as corticosteroids, biologics, plasmapheresis, and novel immunomodulatory therapies. Only studies that reported detailed treatment protocols and outcomes were considered. Exclusion criteria encompassed studies focusing solely on VCA feasibility, anatomy, or surgical techniques without rejection treatment data, as well as non-VCA transplant studies, non-English publications, and systematic reviews or meta-analyses reporting non-original data. All non-peer reviewed studies were excluded. Furthermore, two cases of facial retransplantation were found in literature but not included in qualitative analysis for better comparability amongst other VCA cases (15, 20). In cases where multiple studies reported on the same patient cohort, the most comprehensive publication—detailing immunosuppressive strategies and the longest follow-up—was selected.

**Figure 1:**
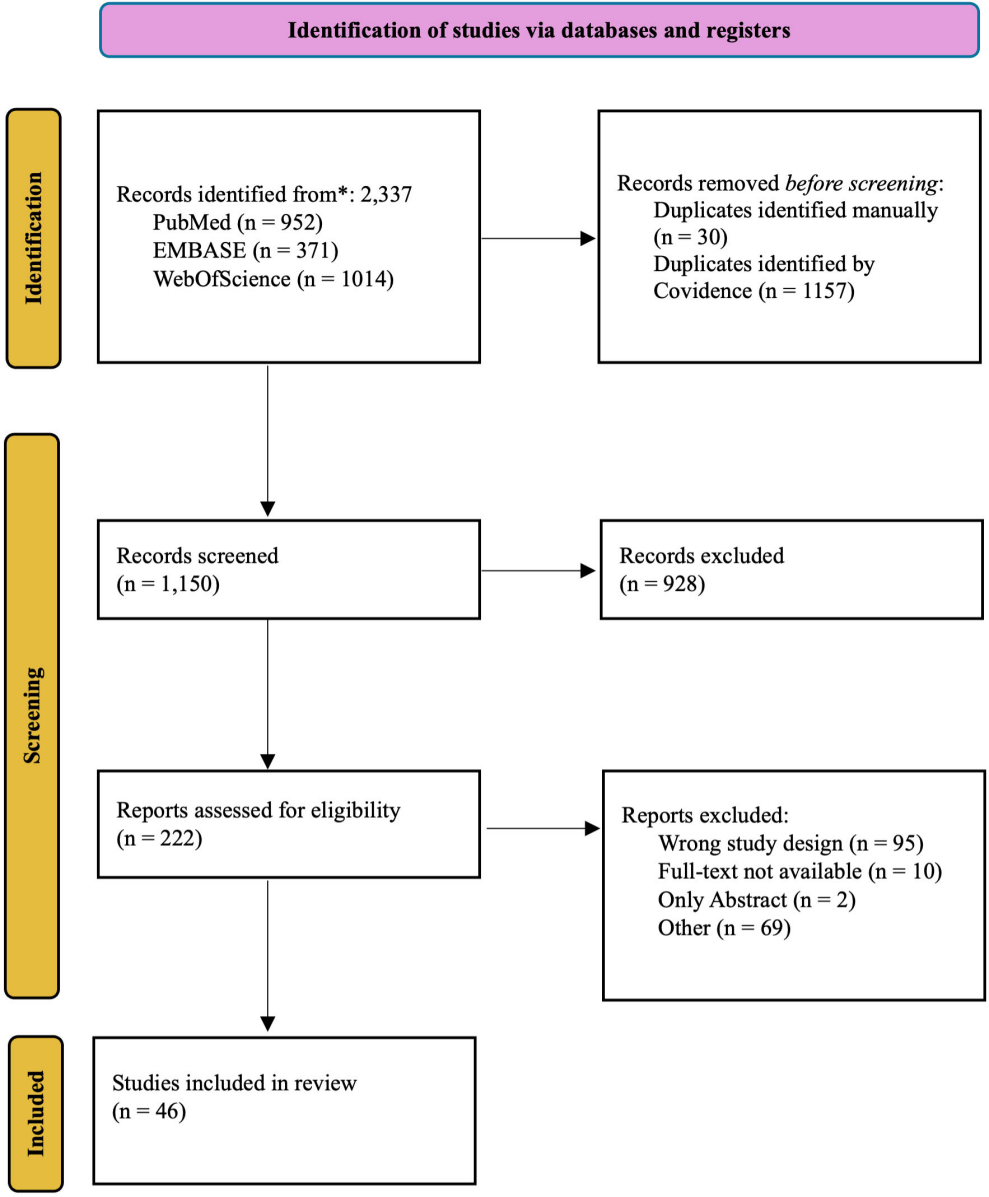
PRISMA 2020 flowchart highlighting study workflow.

Title and abstract screening were independently conducted by two reviewers (T.S. and T.N.), followed by a full-text review of eligible studies. Any discrepancies were resolved through discussion with a third reviewer (L.K.). The full study selection process is outlined in **Figure 1** (PRISMA 2020 flowchart).

### 2.2 Quality assessment

The methodological quality of human studies was evaluated using the Newcastle-Ottawa Scale (NOS) and Level of Evidence (LOE) frameworks. The NOS system assessed three key domains: selection of study cohorts, comparability of study groups, and assessment of outcomes/exposures, with a higher NOS score indicating lower risk of bias. The LOE system ranked studies based on methodological rigor, classifying systematic reviews and randomized controlled trials (RCTs) as LOE I, while retrospective cohort studies were categorized as LOE III-IV. Further quality assessment details are presented in **Supplementary Tables 1, 2**.

### 2.3 Data extraction

In the blinded, dual-review process, the following variables were extracted for human studies: first author, Digital Object Identifier, study title, year of publication, region of publication, institution of transplantation, sample size, recipient sex and age, donor sex and age, type of transplanted VCA, length of follow-up, indication for VCA, induction and maintenance immunosuppressive regimens, presence of rejection (yes/no/acute/chronic), Banff classification of rejection, treatment of rejection episodes (corticosteroid therapy, immunosuppressive modifications, biologic agents, plasmapheresis, extracorporeal photopheresis, donor-specific antibody removal, and adjunct therapies), and overall graft outcome.

Treatment outcomes for acute rejection were categorized as successful if the rejection episode was ultimately reversed and the graft was preserved, regardless of whether multiple lines of therapy or protocol modifications were required. Treatment was defined as unsuccessful only if the rejection episode progressed to total graft loss despite therapeutic intervention. Temporary histological persistence that subsequently resolved with treatment escalation was considered part of a successful management course.

## 3 Results

A total of 1,150 articles were screened, with 46 (4.0%) meeting the inclusion criteria. Due to the limited number of VCAs performed globally and overlapping reports on the same cases, studies were grouped by individual VCA cases. Year of publication spanned from 1999 to 2024. Case reports (n = 25; 54%) and case series (n = 18; 39%) were the most common study types. The mean (± SD) NOS was 5.1 (± 0.3), indicating low to moderate methodological quality.

### 3.1 Patient demographics

Overall, n = 136 (100%) VCA recipients were included. The recipient cohort was predominantly male, with 73% (n = 99) male patients. In donors, n = 51 (38%) were male, whereas gender was not declared in n = 78 (57%) cases. Recipient age ranged from 1 to 69 years, with a mean (± SD) of 39.5 (± 12.9) years. Donor age ranged from 8 to 65 years, with a mean (± SD) of 37.3 (± 12.9) years. The mean (± SD) follow-up period was 35.6 (± 34.4) months and ranged from 1.5 to 228 months. Upper extremity was the most common VCA type (n = 69; 51%), including bilateral procedures in n = 28 cases (21%), followed by face (n = 33; 24%) and abdominal wall transplants (n = 33; 24%) as well as n = 1 (0.7%) case of scalp and penile transplantation, each. More details are provided in **Table 1**.

**Table 1:** Demographical details of patient cohort.

DOI	Author	Title	Year of publication	Region of publication	Study type	Sample size	Recipient age	Recipient sex	Donor age	Donor sex	Length of follow-up	Type of VCA	Indication for VCA
DOI: 10.1016/s0140-6736(99)02062-0	Dubernard et al.	Human hand allograft: report on first 6 months	1999	France	CR	1	48	m	41	m	6 mo	Right distal forearm	Circular saw amputation
DOI: 10.1097/01.SLA.0000078945.70869.82	Dubernard et al.	Functional Results of the First Human Double-Hand Transplantation	2003	France	CR	1	33	m	18	m	15 mo	Bilateral hand allograft	Blast injury
DOI: 10.1016/j.jhsa.2004.05.007	Gabl et al.	Bilateral Hand Transplantation: Bone Healing Under Immunosuppression with Tacrolimus, Mycophenolate Mofetil, and Prednisolone	2004	Austria	CR	1	47	m	N/A	N/A	1 y	Bilateral hand transplant	Bomb explosion
DOI: 10.1097/01.tp.0000168454.68139.0a	Schneeberger et. al.	Cytomegalovirus-Related Complications in Human Hand Transplantation	2005	Austria/USA	CS	18	Mean 32, range: 19-52	m	Mean 33, range: 16-50	male	N/A	Hand allograft	N/A
DOI: 10.1016/S0140-6736(06)68935-6	Devauchelle et al.	First human face allograft: early report	2006	France	CR	1	38	f	46	f	12 w	Facial soft tissue allotransplant	Dog bite
DOI: 10.1111/j.1600-6143.2006.01266.x	Schneeberger et. al.	Status 5 Years after Bilateral Hand Transplantation	2006	Austria/USA	CR	1	48	m	N/A	N/A	5 y	Bilateral hand	Traumatic amputation at wrist level
DOI: 10.1056/NEJMoa072828	Dubernard et al.	Outcomes 18 Months after the First Human Partial Face Transplantation	2007	France	CR	1	38	f	46	f	18 mo	Facial soft tissue allotransplant	Dog bite
DOI: 10.1016/j.jhsa.2008.02.015	Breidenbach et al.	Outcomes of the First 2 American Hand Transplants at 8 and 6 Years Posttransplant	2008	USA	CS	2	P1:37,P2: 36	m	N/A	N/A	8 y and 6 y	Hand transplant (P1: left dominant, P2: left nondominant)	Firecracker accident
DOI: 10.1016/j.main.2008.02.002	Herzberg et al.	Clinical evaluation of two bilateral hand allotransplantations at six- and three-years follow-up	2008	France	CS	2	P1: 33, P2: 21	m	P1: 18, P2: N/A	m	P1: 6 y, P2: 3 y	Hand allograft	P1: blast injury, P2: farm injury
DOI: 10.1111/j.1600-6143.2007.02105.x	Schneeberger et. al.	Atypical Acute Rejection After Hand Transplantation	2008	USA	CS	4	P1: 22, P2: 32, P3: 23, P4: 36	m	N/A	N/A	P1: 57 mo, P2: 65 mo, P3: 9 mo, P4: 73 mo	P1: unilateral hand, P2: unilateral hand, P3: bilateral hand, P4: unilateral hand	Traumatic amputation
DOI: 10.1016/j.surg.2008.06.025	Ravindra et. al.	Hand transplantation in the United States: Experience with 3 patients	2008	USA	CS	3	P1: 37, P2: 36, P3: 54	m	N/A	N/A	N/A	Hand allograft	P1: firecracker accident, amputation at distal forearm, P2: firecracker accident, P3: amputation of dominant right hand in industrial press accident
DOI: 10.1016/j.transproceed.2009.01.013	Brandacher et al.	The Innsbruck Hand Transplant Program: Update at 8 Years First Transplant After the	2009	Austria	CS	3	P1 47, P2: 41, P3: 23	m	N/A	N/A	8 y, 5 y, 2 y	P1 and P3: bilateral hands, P2: bilateral foreram	P1 and P3: explosion, P2: electrical current accident
DOI: 10.1016/j.transproceed.2009.01.020	Selvaggi et. al.	Abdominal Wall Transplantation: Surgical and Immunologic Aspect	2009	USA	CS	14	10 adult, 4 pediatric patients (age range: 1-53)	8m, 6f	N/A	N/A	N/A	Abdominal wall combined with isolated intestine, multivisceral (liver, stomach, pancreas, small bowel), modified multivisceral transplants (multivisceral minus liver graft)	Gardner syndrome (n=5), trauma (n=3), intestinal motility disorders (Hirschsprung disease and intestinal pseudo-obstruction, n=3), gastroschisis (n=2) and Churg-Strauss vasculitis (n=1)
DOI: 10.1016/j.transproceed.2009.01.018	Schneeberger et. al.	Alemtuzumab: Key for Minimization of Maintenance Immunosuppression in Reconstructive Transplantation?	2009	USA	CS	4	P1: 23, P2: 54, P3: 46, P4: 31	P1, P2, P4: m; P3 f	N/A	N/A	P1: 25 mo, P2: 19 mo, P3: 18 mo, P4: 7 mo	Unilateral (P2) or bilateral (P1, P3, P4) hand allografts	Amputations at level of proximal (n=1), mid (n=2) or distal (n=4) forearm
DOI: 10.1097/PRS.0b013e3181c15c4c	Siemionow et. al.	First U.S. Near-Total Human Face Transplantation: A Paradigm Shift for Massive Complex Injuries	2010	USA	CR	1	45	f	N/A	N/A	8 mo	Near-total face	Ballistic trauma
DOI: 10.1097/SLA.0b013e318226a607	Barett et al.	Full Face Transplant: The First Case Report	2011	Spain	CR	1	30 y	m	41 y	m	120 d	All facial soft tissues and underlying bone structures together with vascular (Carotid arteries) and nerve pedicles (sensory branches trigeminal nerve, facial nerve, orbicularis oculi, buccal, zygmoatic nerve)	Ballistic trauma
DOI: 10.1111/j.1600-6143.2011.03503.x	Cavadas et al.	Bilateral Trans-humeral Arm Transplantation: Result at 2 years	2011	Spain	CR	1	29 y	m	25 y	m	2 y	Bilateral forearm	High-voltage electrical injury
DOI: 10.1111/j.1600-6143.2010.03406.x	Lantieri et al.	Feasibility, Reproducibility, Risks and Benefits of Face Transplantation: A Prospective Study of Outcomes	2011	France	CR	4	P1: 29, P3: 27, P4: 37, P5: 33	m	P1: 65, P3:43, P4: 59, P5: 55	m	38 mo	Face	P1: NF1, P4: burn, P3,5: ballistic trauma
DOI: 10.1111/j.1600-6143.2010.03368.x	Pomahac et. al.	Restoration of Facial Form and Function After Severe Disfigurement from Burn Injury by a Composite Facial Allograft	2011	USA	CR	1	59	m	60	m	1 y	Facial transplant	High voltage electrical burn
DOI: 10.1097/TP.0b013e31826c3915	Pei et. al.	A Report of 15 Hand Allotransplantations in 12 Patients and Their Outcomes in China	2012	China	Cohort study	12	P1: 39, P2: 27, P3: 25, P4: 24, P5: 37, P6: 19, P7: 50, P8: 43, P9: 52, P10: 37, P11: 19, P12: 38	m	P1: 29, P2: 25, P3: 30, P4: 29, P5: 35, P6: 20, P7: 48, P8: 35, P9: 50, P10: 42, P11: 24, P12: 23	m	P1: 10 y, P2: 2 y, P3: 1 y, P4: 1 y, P5: 9 y, P6: 8 y, P7: 7 y, P8: 2 y, P9: 6 y, P10: 1 y, P11: 2 y, P12: 2 y	P1: right wrist, P2: right wrist, P3: right wrist, P4: right thumb, P5: double proximal forearm, P6: double wrist, P7: left proximal forearm, P8: right distal forearm, P9: double proximal forearm, P10: right proximal forearm, P11: left palm, P12: right wrist	P1: traumatic amputation, P2: explosion, P3: traumatic amputation, P4: explosion, P5: explosion, P6: cold injury, P7: explosion, P8: explosion, P9: machine injury, P10: machine injury, P11: machine injury, P12: traumatic amputation
DOI: 10.1111/ajt.12715	Chandraker et al.	The Management of Antibody-Mediated Rejection in the First Presensitized Recipient of a Full-Face Allotransplant	2014	USA	CR	1	45	f	45	f	N/A	Face allotransplant	Lye burn
DOI: 10.1016/j.transproceed.2014.08.028	Kaminska et al.	Significant Infections After Hand Transplantation in a Polish Population	2014	Poland	CS	5	P1: 56, P2: 28, P3: 34, P4: 29, P5: 30	4m, 1f	P1: 47, P2: 50, P3: 41, P4: 53, P5: 51	2m, 3f	Up to 74 mo	Hand allograft	N/A	
DOI: 10.1111/ajt.13103	Diaz-Siso et al.	Initial Experience of Dual Maintenance Immunosuppression With Steroid Withdrawal in Vascular Composite Tissue Allotransplantation	2015	USA	CS	5	P1: 59, P2: 25, P3: 30, P4: 57, P5: 65	P1, 2, 3, 5: m, P4: f	N/A	N/A	Median 34 mo, range: 28–58 mo	P1-4: face allotransplant, P5: upper extremity	P1-3: high voltage burn, P4: animal attack, P5: septic shock with bilateral upper extremity amputation
DOI: 10.1155/2015/356459	Kanitakis et al.	Premalignant and Malignant Skin Lesions in Two Recipients of Vascularized Composite Tissue Allografts (Face, Hands)	2015	France	CS	2	P1: 38, P2: 27	f	P1:46, P2: 40	f	P1: 6 y, P2: 8.4 y	P1: partial face allograft, P2: bilateral hand-allograft	P1: dog attack, P2: electrocution
DOI: 10.1371/journal.pone.0136235	Kim et al.	Clonal CD8+ T Cell Persistence and Variable Gene Usage Bias in a Human Transplanted Hand	2015	USA	CR	1	27	f	N/A	N/A	750 d	Hand allograft	N/A
DOI: 10.1097/SAP.0000000000000758	Kuo et al.	The First Hand Allotransplantation in Taiwan A Report at 9 Months	2015	Taiwan	CR	1	45	m	37	m	9 mo	Hand allograft	Traumatic amputation
DOI: 10.1097/TP.0000000000000765	Petruzzo et. al.	Clinicopathological Findings of Chronic Rejection in a Face Grafted Patient	2015	France	CR	1	27	m	N/A	N/A	N/A	Face allotransplant including edentulous mandible, upper and lower lips, cheeks, and chin	Pyrotechnic explosion
DOI: 10.1097/SLA.0000000000000627	Petruzzo et. al.	Outcomes After Bilateral Hand Allotransplantation A Risk/Benefit Ratio Analysis	2015	France	CS	5	P1: 33, P2: 21, P3: 27, P4: 29, P5: 21	P1, 2, 4, 5: m, P3: f	P1: 18, P2: 45, P3: 40, P4: 29, P5: 18	N/A	3 to13 y	Bilateral hand	P1: explosion, P2: crush, P3: electrocution, P4: burning, P5: explosion
DOI: 10.1097/PRS.0000000000002605	Aycart et al.	A Retrospective Analysis of Secondary Revisions after Face Transplantation: Assessment of Outcomes, Safety, and Feasibility	2016	USA	CS	7	P1: 59, P2: 25, P3: 30, P4: 57, P5: 44, P6: 39, P7: 33	P1, 2, 3, 6, 7: m, P4, 5: f	N/A	N/A	Up to 42 mo	Facial allograft including soft tissue, bone and teeth	P1-3: electrical burn, P4: animal attack, P5: chemical burn, P6-7: ballistic trauma
DOI: 10.1097/PRS.0000000000002153	Selber et. al.	Simultaneous Scalp, Skull, Kidney, and Pancreas Transplant from a Single Donor	2016	USA	CR	1	55	m	33	m	1 y	Scalp and skull	Calvaria osteoradionecrosis, resulting in unstable scalp
DOI: 10.1111/ajt.14440	Grahammer et al.	Benefits and limitations of belatacept in 4 hand-transplanted patients	2017	Austria	CS	4	N/A	m	N/A	N/A	P1: 191 d, P2: 13 y, P3: 6 y, P4: N/A	Hand allograft	N/A
DOI: 10.4103/ijps.IJPS_96_17	Iyer et al.	First two bilateral hand transplantations in India (Part 4): Immediate post-operative care, immunosuppression protocol and monitoring	2017	India	CS	2	P1: 31, P2: 31	m	N/A	N/A	1 y	Bilateral hand transplant	N/A
DOI: 10.1002/micr.30272	Özkan et al.	Face allotransplantation for various types of facial disfigurements: a series of five cases.	2017	Turkey	CS	5	P1: 19, P2: 35, P3: 26, P4: 54, P5: 22, mean: 31.2	m	P1: 37, P2: 19, P3: 42, P4: 31, P5: 34	m	Range: 11 mo to 2 y	Face allograft	P1-2: burn, P3-5: ballistic trauma
DOI: 10.1111/ajt.14910	Cendales et al.	*De novo* belatacept in clinical vascularized composite allotransplantation	2018	USA	CR	1	54	m	N/A	m	20 mo	Forearm allograft	Meat grinder accident
DOI: 10.1097/SLA.0000000000002241	Cetrulo et al.	Penis Transplantation First US Experience	2018	USA	CR	1	64	m	27	m	6 mo	Penis	Subtotal penectomy for penile cancer
DOI: 10.1080/23320885.2018.1431047	Fallahian et al.	Eponychial lesions following bilateral upper extremity vascular composite allotransplantation: a case report	2018	USA	CR	1	42	m	Suitable match	Suitable match	3 y	Bilateral upper extremity allograft	Septic shock
DOI: 10.4097/kjae.2018.71.1.66	Kwon et al.	Anesthetic management of the first forearm transplantation in Korea	2018	Korea	CR	1	35	m	49	m	47 d	Forearm transplant	Trauma
DOI: 10.1111/tri.13096	Weissenbacher et. al.	*De novo* donor-specific HLA antibodies after combined intestinal and vascularized composite allotransplantation — a retrospective study	2018	UK	Cohort study	18	Median: 37.5, range: 26-69	11m, 7f	Median: 24.5, range: 8-49	9m, 9f	N/A	Abdominal wall transplant	Intestinal failure: IBD and Pseudomyxoma peritonei
DOI: 10.1097/GOX.0000000000002995	Atia et al.	Synchronous Abdominal Wall and Small-bowel Transplantation: A 1-year Follow-up	2020	USA	CR	1	37	m	13 y	m	1 y	Abdominal wall transplant	High-output small-bowel enterocutaneous fistulas
DOI: 10.1097/PRS.0000000000007890	Govshievich et al.	Face Transplant: Current Update and First Canadian Experience	2020	Canada	CR	1	64	m	Younger match	m	18 mo	Lower two thirds of facial soft tissue, maxilla, mandibula, nose	Ballistic trauma
DOI: 10.1111/tri.13752	Hautz et al.	Long-term outcome after hand and forearm transplantation – a retrospective study	2020	Austria	CS	5	P1: 47, P2: 41, P3: 23, P4: 55, P5: 55	m	N/A	N/A	P1: 19 y, P2: 16 y, P3: 13 y, P4: 7 y, P5:5 y	P1: bilateral distal forearm,P2: bilateral proximal forearm, P3: bilateral mid forearm, P4: unilateral wrist, P5: wrist	P1, P3: explosion, P2: electric current accident, P4: timber machine accident, P5: car accident
DOI: 10.1097/TP.0000000000003241	Roy et. al.	Lymphocytic Vasculitis Associated With Mild Rejection in a Vascularized Composite Allograft Recipient: A Clinicopathological Study	2020	Canada	CR	1	65	m	N/A	N/A	N/A	Facial transplant: inferior orbits, maxilla, mandible, floor of mouth, nose, lower eyelids, all soft tissues of the face;	Ballistic trauma
DOI: 10.1016/j.trim.2021.101377	Azoury et al.	Successful transatlantic bilateral hand transplant in a young female highly sensitized to HLA class II antigens	2021	USA	CR	1	40	f	N/A	N/A	1 y	Hand transplant	unrecoverable tissue ischemia
DOI: 10.1055/a-2059-5570	Lee et al.	One Year Experience of the Hand Allotransplantation First Performed after Korea Organ Transplantation Act (21) Amendment	2023	South Korea	CR	1	62	m	N/A	N/A	1 y	Hand	Traumatic amputation
DOI: 10.1016/j.ajt.2023.01.016	Murakami et al.	Low-dose interleukin-2 promotes immune regulation in face transplantation: A pilot study	2023	USA	CS	2	P1: 57, P2: 60	P1: f, P2: m	N/A	N/A	48 w	Face	P1: animal attack, P2: N/A
DOI: 10.3389/frtra.2024.1339898	Zaccardelli et. al.	Case Report: Post-transplant lymphoproliferative disorder as a serious complication of vascularized composite allotransplantation	2024	USA	CR	1	65	m	N/A	N/A	12 y	Bilateral upper extremity	Bilateral upper (below-elbow) and lower extremity (below-knee) amputation secondary to urosepsis complicated by ARDS

CR, Case Report; CS, Case Series; STR, Steroid Therapy; MPED, Methylprednisolone; PDN, Prednisone; ATG, Anti-Thymocyte Globulin; MMF, Mycophenolate Mofetil; TAC, Tacrolimus; HLA, Human Leukocyte Antigen; CAMR, Chronic Antibody-Mediated Rejection; VCA, Vascularized Composite Allotransplantation; AR, Acute Rejection; CR, Chronic Rejection; POD, Post-Operative Day; POM, Post-Operative Month; POY, Post-Operative Year; mo, months; y: year(s); w, week(s); d, day(s); N/A, Not Applicable.

### 3.2 Indications for VCA

The most common indication for VCA was trauma (n = 53; 39%), including n = 13 (9.6%) ballistic injuries, followed by gastrointestinal disorders (n = 30; 22%), such as Gardner Syndrome (n = 5; 3.7%) and Hirschsprung disease (n = 3; 2.2%). Here, VCA was typically required due to abdominal wall failure following repeated surgical intervention. Burn injuries accounted for n = 20 (15%) VCAs, including n = 12 (8.8%) electrical burns. Other indications included amputations (n = 7; 5.1%), animal bites (n = 6; 4.4%), and conditions such as osteoradionecrosis (n = 1; 0.7%), neurofibromatosis type I (n = 1; 0.7%), or irreversible tissue ischemia (n = 1; 0.7%). Further information is provided in **Table 1**.

### 3.3 Immunosuppressive regimens

Induction therapy included antithymocyte globulin (ATG) in n = 74 (46%) cases, followed by MMF in n = 49 cases (31%) and TAC in n = 48 cases (30%). Steroids (STR) were administered in n = 66 cases (42%), primarily as prednisone (n = 35; 22%) and methylprednisolone (n = 24; 15.0%). Further induction agents included alemtuzumab (n = 45; 28%), basiliximab (n = 16; 10%), and belatacept (n = 4; 2.5%), with smaller numbers receiving cyclophosphamide, azathioprine, donor bone marrow cells (each n = 2; 1.3%), and rituximab (n = 1; 0.6%).

**Figure 2:**
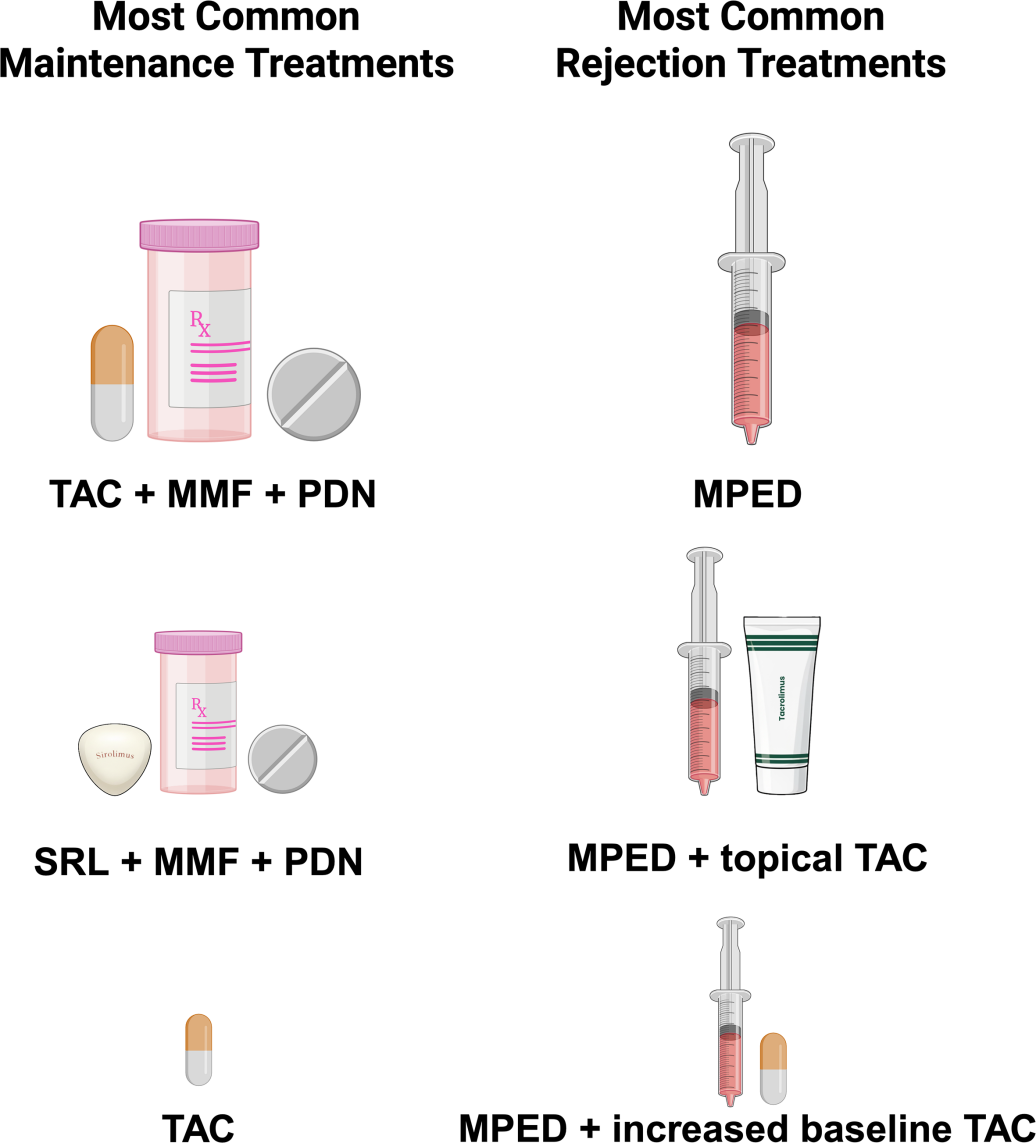
Drug combinations for graft maintenance and acute rejection treatment. Legend: The most common maintenance immunosuppression combinations were TAC + MMF + PDN (48 treatments), followed by SRL + MMF + PDN (4 treatments) and TAC monotherapy (3 treatments). The most common drug combination in acute rejection treatment was MPED Pulse/Bolus monotherapy (42 episodes), followed by MPED + topical TAC (26 episodes) and MPED with an increase in baseline TAC (25 episodes). TAC, Tacrolimus; MMF, Mycophenolate Mofetil; PDN, Prednisone; SRL, Sirolimus; MPED, Methylprednisolone.

Maintenance therapy varied from induction in dosage and drug composition. It predominantly included TAC (n = 133; 98%), STR (n = 94; 69%), and MMF (n = 92; 68%). The most common STR was prednisone (n = 63; 46%). Further maintenance agents were sirolimus (n = 15; 11%), azathioprine (n = 4; 2.9%), belatacept (n = 4; 2.9%), everolimus (n = 2; 1.5%), extracorporeal photopheresis (n = 4; 2.9%), extracorporeal photochemotherapy (n = 2; 1.5%), basiliximab (n = 1; 0.7%), and IL-2 therapy (n = 2; 1.5%). Further details are provided in **Table 2**, **Figure 2**.

**Table 2:** Immunosuppressive baseline regimen of patient cohort.

DOI	Author	Title	Year of publication	Induction immunotherapy	Maintenance immunotherapy
DOI: 10.1016/s0140-6736(99)02062-0	Dubernard et al.	Human hand allograft: report on first 6 months	1999	ATG 75 mg/day × 10 days, tacrolimus (10–15 ng/mL), mycophenolic acid 2 g/day, prednisone tapered from 250 mg (day 1) to 20 mg/day, CD25 monoclonal antibody on days 26 and 100 post-transplant	Tacrolimus (5–10 ng/mL), mycophenolic acid 2 g/day, prednisone 20 mg at 3 months, 15 mg at 6 months
DOI: 10.1097/01.SLA.0000078945.70869.82	Dubernard et al.	Functional Results of the First Human Double-Hand Transplantation	2003	ATG 1.25 mg/kg/day × 10 days (6 h infusion); tacrolimus 0.2 mg/kg/day (15–20 ng/mL), prednisone 250 mg on day 1, 1 mg/kg/day × 10 days then tapered to 20 mg/day, MMF 2 g/day	Prednisone 10 mg/day, tacrolimus (5–10 ng/mL), MMF 2 g/day
DOI: 10.1016/j.jhsa.2004.05.007	Gabl et al.	Bilateral Hand Transplantation: Bone Healing Under Immunosuppression with Tacrolimus, Mycophenolate Mofetil, and Prednisolone	2004	ATG 2.5 mg/kg × 4 days (started during surgery); methylprednisolone 500 mg i.v. pre-revascularization, then 250 mg on day 1, 125 mg on day 2; switched to oral prednisolone tapered to 25 mg by day 8	Prednisolone reduced to 7.5 mg at 1 year; tacrolimus started at 0.2 mg/kg, adjusted (15 ng/mL first month, 12 ng/mL 2–6 months, 10 ng/mL later), MMF 1 g BID
DOI: 10.1097/01.tp.0000168454.68139.0a	Schneeberger et. al.	Cytomegalovirus-Related Complications in Human Hand Transplantation	2005	Four protocols: 1) ATG + MMF + tacrolimus + steroids; 2) IL-2 receptor antagonists + MMF + tacrolimus + steroids; 3) MMF + tacrolimus + steroids; 4) ATG + MMF + cyclosporine A + steroids. In CMV cohort: ATG 2.5 mg/kg × 4 days or basiliximab 20 mg 2 h pre-op, day 4, and day 45	CNI (CyA, FK506), MMF, steroids (89%); some switched to sirolimus, others topical tacrolimus or steroids
DOI: 10.1016/S0140-6736(06)68935-6	Devauchelle et al.	First human face allograft: early report	2006	Thymoglobulin 1.25 mg/kg/day × 10 days, tacrolimus (10–15 ng/mL), MMF 2 g/day, prednisone (250 mg day 1, 100 mg day 2, 60 mg/day × 10 days then tapered to 5 mg/day), aspirin and heparin. Frozen bone marrow infused on days 4 and 11 post-transplant (nucleated cells: 1.6–1.8×10^8^/kg; CFU-GM: 2–4×10^4^/kg; CD34^+^: 0.12×10^6^/kg; CD3^+^: 2.7–4.1×10^6^/kg)	MMF, tacrolimus, prednisone, topical tacrolimus/steroids
DOI: 10.1111/j.1600-6143.2006.01266.x	Schneeberger et. al.	Status 5 Years after Bilateral Hand Transplantation	2006	ATG	Tacrolimus (10 ng/mL), MMF 2 g/day, prednisone 5 mg/day; sirolimus added after 30 months, steroids withdrawn, tacrolimus stopped 3 months later
DOI: 10.1056/NEJMoa072828	Dubernard et al.	Outcomes 18 Months after the First Human Partial Face Transplantation	2007	Thymoglobulin i.v. × 10 days, tacrolimus (10–15 ng/mL), MMF 2 g/day, prednisone (250 mg day 1, 100 mg day 2, 60 mg/day through day 12, then tapered)	Sirolimus introduced at 11 months, tacrolimus/sirolimus stopped 5 weeks later due to nephrotoxicity; sirolimus reintroduced (8–12 ng/mL), MMF 2 g/day, prednisone 10 mg/day
DOI: 10.1016/j.jhsa.2008.02.015	Breidenbach et al.	Outcomes of the First 2 American Hand Transplants at 8 and 6 Years Posttransplant	2008	Anti- IL25R antibody, basiliximab	Tacrolimus, MMF, prednisone; in P2, MMF switched to rapamycin after 4 weeks
DOI: 10.1016/j.main.2008.02.002	Herzberg et al.	Clinical evaluation of two bilateral hand allotransplantations at six- and three-years follow-up	2008	P1: Induction with polyclonal antilymphocyte antibodies, tacrolimus, prednisolone, and MMF × 10 days; P2: Same as P1	Tacrolimus, prednisone, MMF
DOI: 10.1111/j.1600-6143.2007.02105.x	Schneeberger et. al.	Atypical Acute Rejection After Hand Transplantation	2008	P1: ATG; P2: Basiliximab; P3: Alemtuzumab; P4: Basiliximab;	P1: Tacrolimus, MMF, steroids; after AR: MMF increased to 2 g/day, prednisone to 8 mg/day, tacrolimus 3 mg BID, methylprednisolone 16 mg/day; P2–3: Tac, MMF, steroids; P4: rapamycin, MMF, steroids
DOI: 10.1016/j.surg.2008.06.025	Ravindra et. al.	Hand transplantation in the United States: Experience with 3 patients	2008	P1: Basiliximab 20 mg i.v. pre-op and day 4; P2: Basiliximab 20 mg i.v. pre-op and day 4; P3: Alemtuzumab 30 mg single intra-op dose	P1: Tac (15–20 ng/mL first 6 months), MMF 1 g BID, prednisone 10 mg/day at 3 months, tapered to 7.5 mg/day at 6 months; P2–3: Tac, MMF, steroids; P3: peri-op methylprednisolone for 3 days
DOI: 10.1016/j.transproceed.2009.01.013	Brandacher et al.	The Innsbruck Hand Transplant Program: Update at 8 Years First Transplant After the	2009	P1, P2: ATG; P3: Alemtuzumab	P1–2: Tac, MMF, prednisone; planned switch to sirolimus/everolimus
DOI: 10.1016/j.transproceed.2009.01.020	Selvaggi et. al.	Abdominal Wall Transplantation: Surgical and Immunologic Aspect	2009	Alemtuzumab	Steroid-free tacrolimus-based therapy
DOI: 10.1016/j.transproceed.2009.01.018	Schneeberger et. al.	Alemtuzumab: Key for Minimization of Maintenance Immunosuppression in Reconstructive Transplantation?	2009	P1: Alemtuzumab 2 doses at 20mg; P2: Alemtuzumab 30mg i.v.	P1: Tac, steroids; MMF added after AR; P2: Tac (10–15 ng/mL) + MMF; MMF stopped/restarted for CMV; P3: Tac, MMF, steroids; P4: Tac switched to sirolimus
DOI: 10.1097/PRS.0b013e3181c15c4c	Siemionow et. al.	First U.S. Near-Total Human Face Transplantation: A Paradigm Shift for Massive Complex Injuries	2010	ATG	Corticosteroids, tacrolimus, MMF
DOI: 10.1097/SLA.0b013e318226a607	Barett et al.	Full Face Transplant: The First Case Report	2011	Slow infusion thymoglobulin 2 mg/kg 2 h pre-op, prednisone 1 g	Prednisone tapered to 10 mg/day, tacrolimus (10–15 ng/mL), MMF 2 g/day
DOI: 10.1111/j.1600-6143.2011.03503.x	Cavadas et al.	Bilateral Trans-humeral Arm Transplantation: Result at 2 years	2011	Alemtuzumab 30 mg i.v., methylprednisolone 500 mg and 250 mg on days 1 and 2, then stopped	Tacrolimus, MMF 2 g/day; tacrolimus switched to sirolimus at POD 332
DOI: 10.1111/j.1600-6143.2010.03406.x	Lantieri et al.	Feasibility, Reproducibility, Risks and Benefits of Face Transplantation: A Prospective Study of Outcomes	2011	ATG (1 mg/kg/day × 10 days), tacrolimus (10–13 ng/mL for 3 months), MMF 2 g/day (AUC 40–60 ng/mL), prednisone (500 mg day 1, 250 mg day 2, 120 mg day 3, then 60 mg/day × 7 days, tapered to 10 mg/day)	Tacrolimus (8–10 ng/mL), MMF, prednisone; ECP for P3–5
DOI: 10.1111/j.1600-6143.2010.03368.x	Pomahac et. al.	Restoration of Facial Form and Function After Severe Disfigurement from Burn Injury by a Composite Facial Allograft	2011	500 mg methylprednisolone and r-ATG 1.5 mg/kg pre-reperfusion; 1000 mg MMF pre-surgery	Prednisolone 15–30 mg/day, MMF 2 g/day (switched to mycophenolic acid 720–1120 mg/day), tacrolimus 6–12 mg/day (10 ng/mL trough); steroid boluses, topical clobetasol and tacrolimus
DOI: 10.1097/TP.0b013e31826c3915	Pei et. al.	A Report of 15 Hand Allotransplantations in 12 Patients and Their Outcomes in China	2012	P1: ATG 100 mg/day + tacrolimus 5 mg/day + MMF 750 mg/day + MPED 1 g/day; P2: Same as P1; P3: CTX 400 mg/day; P4: CTX 400 mg/day; P5: ATG 100 mg/day + tacrolimus + MMF 500 mg/day + MPED 1 g/day; P6: ATG 80 mg/day + tacrolimus 5 + MPED 800 mg/day; P7: Same as P6; P8: Same as P6; P9: ATG 80 mg/day + tacrolimus 5 mg/day + MPED 800 mg/day; P10–12: Data not available	P1: Tac 3 mg/day, MMF (stopped at 6 months), pred 5 mg/day; P3–9: Tac 1–3 mg/day, MMF 1 g/day, pred 5–10 mg/day; P2/10–12: data N/A
DOI: 10.1111/ajt.12715	Chandraker et al.	The Management of Antibody-Mediated Rejection in the First Presensitized Recipient of a Full-Face Allotransplant	2014	ATG 1.5 mg/kg/day × 4 days; MMF 1 g i.v. BID; steroid taper; tacrolimus 2 mg BID (goal 10 ng/mL). In high-risk patients, plasmapheresis every other day from POD1, each followed by 10 g IVIG (150 mg/kg). Post-op immunosuppression tailored by biopsy and DSA	MMF, tacrolimus
DOI: 10.1016/j.transproceed.2014.08.028	Kaminska et al.	Significant Infections After Hand Transplantation in a Polish Population	2014	Basiliximab	Tacrolimus (10–15 ng/mL), MMF 2 g/day, steroids (20–40 mg/day)
DOI: 10.1111/ajt.13103	Diaz-Siso et al.	Initial Experience of Dual Maintenance Immunosuppression With Steroid Withdrawal in Vascular Composite Tissue Allotransplantation	2015	ATG 1.5 mg/kg/day × 4 days, methylprednisolone 500 mg/day then tapered, MMF 1000 mg pre-surgery	Tacrolimus (10–15 ng/mL days 3–21), MMF 1 g BID, prednisone taper (20 mg on day 5)
DOI: 10.1155/2015/356459	Kanitakis et al.	Premalignant and Malignant Skin Lesions in Two Recipients of Vascularized Composite Tissue Allografts (Face, Hands)	2015	P1: Induction: tacrolimus, MMF, prednisone, ATG; donor bone marrow cells infused on days 4 and 11 post-transplant. P2: N/A	P1: Sirolimus, MMF, prednisone (SRL introduced at 11 months); P2: Steroids, MMF, tacrolimus
DOI: 10.1371/journal.pone.0136235	Kim et al.	Clonal CD8+ T Cell Persistence and Variable Gene Usage Bias in a Human Transplanted Hand	2015	N/A	TAC, MMF, prednisone, everolimus
DOI: 10.1097/SAP.0000000000000758	Kuo et al.	The First Hand Allotransplantation in Taiwan A Report at 9 Months	2015	ATG 1.25 mg/kg/day × 10 days starting intraop, methylprednisolone 500 mg pre-anesthesia, 250 mg post-ATG, 125 mg on POD1, then tapered to 10 mg. Tacrolimus started on day 1 (10–15 ng/mL first 6 months, 8–10 ng/mL after, then 5–8 ng/mL)	Prednisone 10 mg/day, tacrolimus, MMF 2 g/day
DOI: 10.1097/TP.0000000000000765	Petruzzo et. al.	Clinicopathological Findings of Chronic Rejection in a Face Grafted Patient	2015	ATG	Steroids 5 mg/day, tacrolimus (5–10 ng/mL), MMF 2 g/day; POD4 donor bone marrow infusion. Current: everolimus 3 mg/day, steroids 16 mg/day; extracorporeal photochemotherapy
DOI: 10.1097/SLA.0000000000000627	Petruzzo et. al.	Outcomes After Bilateral Hand Allotransplantation A Risk/Benefit Ratio Analysis	2015	ATG 1.25 mg/kg/day × 10 days. Others: thymoglobulin 3 mg/kg day 1, 2 mg/kg day 2, 1.5 mg/kg/day × 4 days; prednisolone 250 mg day 1, 1 mg/kg/day × 10 days then tapered to 20 mg/day; tacrolimus 0.1 mg/kg/day from day 2 (10–15 ng/mL), MMF 2 g/day	Prednisone 5 mg/day, tacrolimus (5–10 ng/mL), MMF 2 g/day; patient 3: switched to sirolimus and MMF 1 g/day due to AR; patient 5: switched to sirolimus for 14 months due to creatinine rise
DOI: 10.1097/PRS.0000000000002605	Aycart et al.	A Retrospective Analysis of Secondary Revisions after Face Transplantation: Assessment of Outcomes, Safety, and Feasibility	2016	N/A	MMF 1 g BID, tacrolimus (10–15 ng/mL), prednisone taper to 20 mg
DOI: 10.1097/PRS.0000000000002153	Selber et. al.	Simultaneous Scalp, Skull, Kidney, and Pancreas Transplant from a Single Donor	2016	5 doses rabbit ATG (total 7.14 mg/kg), 3 doses of 500 mg i.v. methylprednisolone	Tacrolimus target 10 ng/mL, MMF 1 g BID, prednisone 5 mg/day, topical tacrolimus added
DOI: 10.1111/ajt.14440	Grahammer et al.	Benefits and limitations of belatacept in 4 hand-transplanted patients	2017	Belatacept 5 mg/kg i.v. every 2 weeks for 5 doses, then every 4 weeks	P1: Tacrolimus reduced from 8–10 to 5 ng/mL over 6 months; no rejection. P2: Tac 4–5 ng/mL after belatacept added at 13 years. P3: Tac 6–8 ng/mL, rapamycin 8–10 ng/mL, pred 5 mg/day, improved 4 months after belatacept. P4: Tac 8 ng/mL, MMF 1 g/day, pred 7.5 mg/day; belatacept started
DOI: 10.4103/ijps.IJPS_96_17	Iyer et al.	First two bilateral hand transplantations in India (Part 4): Immediate post-operative care, immunosuppression protocol and monitoring	2017	Thymoglobulin 1.5 mg/kg i.v.; methylprednisolone 500 mg i.v. stat; tacrolimus 0.05 mg/kg stat; MMF 1000 mg stat. Day 0: tacrolimus 0.1 mg/kg BID, methylprednisolone 250 mg i.v., thymoglobulin 1.5 mg/kg i.v., MMF 1000 mg BID. Days 1–5: thymoglobulin 1.5 mg/kg i.v. × 3 days, prednisolone 0.5 mg/kg/day, tacrolimus 0.1 mg/kg BID, MMF 1000 mg BID	Prednisolone 0.5 mg/kg/day, tacrolimus dose adjusted to levels, MMF 1 g BID
DOI: 10.1002/micr.30272	Özkan et al.	Face allotransplantation for various types of facial disfigurements: a series of five cases.	2017	ATG 2.5 mg/kg/day started intra-op; prednisolone 1000 mg day 0, tapered to 20 mg by week 1. Tacrolimus 0.2 mg/kg/day (15–20 ng/mL) started day 4; ATG stopped days 7–10 based on tacrolimus levels	Prednisolone tapered from 20 to 10 mg/day (6 months), tacrolimus 15–20 ng/mL (3 months), 7–10 ng/mL (6 months), MMF 2 g/day; patient 4 modified due to complications
DOI: 10.1111/ajt.14910	Cendales et al.	*De novo* belatacept in clinical vascularized composite allotransplantation	2018	ATG 1.5 mg/kg × 3 doses	Belatacept 10 mg/kg ×2, then 5 mg/kg; tacrolimus 10–15 ng/mL switched to sirolimus 8–12 ng/mL at 6 months; MMF 1 g BID, prednisone taper to 10 mg. Current: belatacept 5 mg/kg monthly, MMF 500 mg BID, prednisone 10 mg
DOI: 10.1097/SLA.0000000000002241	Cetrulo et al.	Penis Transplantation First US Experience	2018	ATG, MMF, methylprednisolone	MMF, tacrolimus, prednisone taper
DOI: 10.1080/23320885.2018.1431047	Fallahian et al.	Eponychial lesions following bilateral upper extremity vascular composite allotransplantation: a case report	2018	ATG 1.5 mg/kg	Discharge: tacrolimus 8–10 ng/mL, mycophenolate sodium 720 mg BID, prednisone 10 mg/day
DOI: 10.4097/kjae.2018.71.1.66	Kwon et al.	Anesthetic management of the first forearm transplantation in Korea	2018	Basiliximab (20 mg)	Methylprednisolone 125 mg, MMF 750 mg, tacrolimus 5 mg
DOI: 10.1111/tri.13096	Weissenbacher et. al.	*De novo* donor-specific HLA antibodies after combined intestinal and vascularized composite allotransplantation — a retrospective study	2018	Alemtuzumab (30mg i.v.) × 2 doses within 24h	Tacrolimus monotherapy: 10–12 ng/mL (6 months), 8–10 ng/mL after
DOI: 10.1097/GOX.0000000000002995	Atia et al.	Synchronous Abdominal Wall and Small-bowel Transplantation: A 1-year Follow-up	2020	ATG (1.5 mg/kg × 4 doses)	Tacrolimus (15–18 ng/mL ×3 months), MMF 1 g BID, prednisone 20 mg/day taper
DOI: 10.1097/PRS.0000000000007890	Govshievich et al.	Face Transplant: Current Update and First Canadian Experience	2020	ATG, tacrolimus (10–15 ng/mL), MMF, i.v. Solu-Medrol	Maintenance via gastrostomy: prednisone tapered over 5 weeks, MMF same dose, tacrolimus 10–15 μg/L first 6 months, lowered to 8 μg/L at week 34
DOI: 10.1111/tri.13752	Hautz et al.	Long-term outcome after hand and forearm transplantation – a retrospective study	2020	P1, 2: ATG; P3-5: Alemtuzumab	P1,2,3,5: Tacrolimus, MMF, steroids; P4: Tacrolimus,MMF; P5: Belatacept; P2,3: Belatacept
DOI: 10.1097/TP.0000000000003241	Roy et. al.	Lymphocytic Vasculitis Associated With Mild Rejection in a Vascularized Composite Allograft Recipient: A Clinicopathological Study	2020	ATG 10 mg/kg i.v., tacrolimus, MMF 1 g i.v. BID, solumedrol 50 mg i.v. daily tapered to 25 mg	Tacrolimus 10–15 μg/L first 6 months, lowered to 8 μg/L at week 34, MMF 1 g BID, solumedrol 50 mg i.v. tapered to 25 mg; topical tacrolimus added after week 10; basiliximab added monthly (4 doses)
DOI: 10.1016/j.trim.2021.101377	Azoury et al.	Successful transatlantic bilateral hand transplant in a young female highly sensitized to HLA class II antigens	2021	ATG 75 mg × 5 doses	Tacrolimus, MMF 1 g/day, prednisone, rapamycin
DOI: 10.1055/a-2059-5570	Lee et al.	One Year Experience of the Hand Allotransplantation First Performed after Korea Organ Transplantation Act (21) Amendment	2023	Triple induction: tacrolimus (3 mg pre-op, 4 mg/day; target trough 6–8 ng/mL), steroids (500 mg i.v. pre- and post-reperfusion), basiliximab 20 mg i.v. pre-op and day 4 post-op (standard kidney transplant protocol)	Tacrolimus target 6–8 ng/mL, steroids tapered to 10 mg by day 17, MMF 1 g/day from day 14 onwards
DOI: 10.1016/j.ajt.2023.01.016	Murakami et al.	Low-dose interleukin-2 promotes immune regulation in face transplantation: A pilot study	2023	ATG	P1: Tacrolimus, MMF (prednisolone stopped at 4.5 months); P2: Tacrolimus (6–8 ng/mL), MMF 1.5 g/day, pred 5 mg/day, IL-2 protocol
DOI: 10.3389/frtra.2024.1339898	Zaccardelli et. al.	Case Report: Post-transplant lymphoproliferative disorder as a serious complication of vascularized composite allotransplantation	2024	ATG × 4 doses i.v.	Tacrolimus (10–15 ng/mL), MMF 1 g BID, prednisone 7.5 mg/day; tacrolimus and MMF weaned to 5 ng/mL and 360 mg BID

ATG, antithymocyte globulin; AZA, azathioprine; BID, twice daily; CFU-GM, colony-forming-unit granulomacrophage; CyA, cyclosporine A; DSA, donor-specific antibodies; ECP, extracorporeal photopheresis; FK506, tacrolimus; HTN, hypertension; i.v., intravenous; IL-2, interleukin-2; IVIG, intravenous immunoglobulin; MMF, mycophenolate mofetil; MPED, methylprednisolone; mTOR, mammalian target of rapamycin; N/A, not applicable; P, patient; POD, postoperative day; POM, postoperative month; POY, postoperative year; SIR, sirolimus (rapamycin); SRL, sirolimus (rapamycin); Tac, tacrolimus; TPE, therapeutic plasma exchange.

*Frequency, Symptoms and Treatment of Acute Rejection Episodes*.

A total of n = 219 rejection episodes were reported, of which n = 218 were reported as acute. Most patients experienced one (n = 40; 29%), two (n = 19; 14%), or three (n = 17; 13%) rejection episodes. A small subset had four to six rejection episodes (n = 4 each; 2.9%). In selected cases, seven (n = 3; 2.2%) or more than eight rejection episodes (n = 2; 1.5%) were reported. Most commonly, rejection first occurred later than postoperative week (POW) 52 (n = 52), followed by POW 5-12 (n = 42) and POW 13-52 (n = 30). Early rejection within the first four weeks was observed in n = 13 cases (22).

Banff grade I rejection was reported in n = 49 (36%) cases. However, Banff grade II (n = 73; 54%) and grade III (n = 50; 37%) were the most frequent. Banff grade IV rejection was reported in n = 1 (0.7%) case.

Symptoms of acute rejection included skin lesions (n = 43; 32%), edema/swelling (n = 32; 24%), erythema (n = 29; 21%), and rashes (n = 15; 11%). Further signs were sensory changes such as numbness (n = 4; 2.9%), tingling (n = 5; 3.7%), burning sensations (n = 5; 3.7%), or pain (n = 7; 5.1%).

Overall, STR were the mainstay of acute rejection treatment, administered in n = 98 (72%) cases, with methylprednisolone (MPED) (n = 31; 23%), clobetasol (n = 15; 11%), and prednisone (PDN) (n = 11; 8.1%) being the most common agents. TAC was administered in n = 49 (36%) cases, with n = 29 (21%) receiving topical applications. Further immunosuppressive therapies included ATG (n = 19; 14%), alemtuzumab (n = 11; 8.1%), MMF (n = 11; 8.1%), and rituximab (n = 6; 4.4%), while basiliximab (n = 4; 2.9%), sirolimus (n = 2; 1.5%), and plasmapheresis (n = 4; 2.9%) were used in selected cases. Additional agents such as immunoadsorption (n = 3; 2.2%), extracorporeal photochemotherapy (n = 2; 1.5%), bortezomib (n = 1; 0.7%), eculizumab (n = 1; 0.7%), and pimecrolimus (n = 1; 0.7%) were employed. Details on individualized rejection therapies are depicted in **Table 3** as well as data on frequency of different drug regimens in **Figure 2**.

**Table 3:** Rejection frequency, Banff classification, signs of rejection and treatment of rejection in patient cohort.

DOI	Author	Title	Year of publication	Rejection	Banff classification	Signs of rejection	Treatment of rejection
DOI: 10.1016/s0140-6736(99)02062-0	Dubernard et al.	Human hand allograft: report on first 6 months	1999	POW 8–9: Rejection at weeks 8–9	N/A	Mild erythema, dense perivascular mononuclear infiltrate	Increased prednisone (20→40 mg/day), topical tacrolimus/clobetasol, tacrolimus (6→14 mg/day)
DOI: 10.1097/01.SLA.0000078945.70869.82	Dubernard et al.	Functional Results of the First Human Double-Hand Transplantation	2003	POD 53, 82: Two skin rejection episodes	N/A	Mononuclear infiltrate at POD 8, maculopapular forearm lesions, dense dermal infiltrate	Prednisone (40→20 mg/day over 8 days), topical clobetasol, resolved in 10 days
DOI: 10.1016/j.jhsa.2004.05.007	Gabl et al.	Bilateral Hand Transplantation: Bone Healing Under Immunosuppression with Tacrolimus, Mycophenolate Mofetil, and Prednisolone	2004	POW 8: Rejection at week 8	N/A	N/A	Methylprednisolone 750 mg + 2 doses of 500 mg i.v., topical tacrolimus/methylprednisolone
DOI: 10.1097/01.tp.0000168454.68139.0a	Schneeberger et. al.	Cytomegalovirus-Related Complications in Human Hand Transplantation	2005	P1 had AR at POD 34 and 78; P2 at POD 70, 93, 128; P3 at POD 27; P4 had AR at POD 10, 46, 95; 4 acute reactions, 14 without	N/A	Biopsy showed T-cell infiltrate	Tacrolimus, corticosteroids, flumix ointment; severe: steroids, ATG, Campath-1H; CMV: methylpred (250–500 mg ×3), ATG, local steroids
DOI: 10.1016/S0140-6736(06)68935-6	Devauchelle et al.	First human face allograft: early report	2006	POD 18–24: Mucosa and skin rejection	Banff I and II	Diffuse erythema, edema, dense mononuclear infiltrate, apoptotic keratinocytes	Prednisone (25→60 mg/day), tacrolimus (10→15 mg/day), MMF (2→3 g/day), topical clobetazol/tacrolimus
DOI: 10.1111/j.1600-6143.2006.01266.x	Schneeberger et. al.	Status 5 Years after Bilateral Hand Transplantation	2006	POD 55, 188; month 48: Three AR episodes	Banff II	Maculopapular lesions, diffuse lymphocytic/eosinophilic infiltrates, interface dermatitis	Bolus steroids, tacrolimus trough 3–4 ng/mL
DOI: 10.1056/NEJMoa072828	Dubernard et al.	Outcomes 18 Months after the First Human Partial Face Transplantation	2007	POD 18, 214: Two acute rejection episodes	Banff I and II	Erythema, edema on mucosa and skin; lymphocytic infiltrates, keratinocyte apoptosis, CD4+ predominant	Oral prednisone/tacrolimus/MMF increases, clobetasol/tacrolimus topically, methylprednisolone pulses (1000 mg ×3), 750 mg ×3 for 2nd AR
DOI: 10.1016/j.jhsa.2008.02.015	Breidenbach et al.	Outcomes of the First 2 American Hand Transplants at 8 and 6 Years Posttransplant	2008	P1: 3 rejections on days 51, 143, 204; P2: rejection at 5 years	N/A	Rashes	Rabbit ATG, methylprednisone boluses
DOI: 10.1016/j.main.2008.02.002	Herzberg et al.	Clinical evaluation of two bilateral hand allotransplantations at six- and three-years follow-up	2008	P1 POD 53, 82; P2 POD 60, 90	N/A	Pink macules, erythematous papules	Systemic prednisolone increase and topical steroids/tacrolimus
DOI: 10.1111/j.1600-6143.2007.02105.x	Schneeberger et. al.	Atypical Acute Rejection After Hand Transplantation	2008	P1: POM 43; P2: POM 3, POM 27; P3: POD 51, POD 60; P4: POD 10, POD 21, POD 50, POD 77	Banff I to III	Biopsy/histology: Palmar rash, nail loss, CD3+/CD20+/CD79a+ infiltration, perivascular lymphocytes	P1: Methylprednisolone 500 mg i.v. (3d), topical diprosone/tacrolimus; 2nd AR: methylprednisolone 500 mg/d (3d), 2nd course with ATG; P2: steroids (500 mg/d ×2, 250 mg/d ×1, 125 mg/d ×1), topical tacrolimus 14d; P3: solumedrol 500 mg/d (3d), 2nd AR resistant to steroids, alemtuzumab (20 mg); P4: 1st AR: methylprednisolone/topical TAC/clobetasol, 2nd AR: prednisolone 100 mg/d (4d), 3rd AR: rabbit ATG, 4th AR: topical TAC/clobetasol
DOI: 10.1016/j.surg.2008.06.025	Ravindra et. al.	Hand transplantation in the United States: Experience with 3 patients	2008	P1: 3 AR episodes in year 1 (at 2, 5, and 7 months); P2: 5 AR episodes in year 1 and 1 episode in year 5; P3: AR of hand	Banff I to III	N/A	P1: methylprednisolone pulses for all AR; P2: 1st year ARs with i.v. methylprednisolone, 5th year AR: thymoglobulin (6d); P3: topical TAC/clobetasol
DOI: 10.1016/j.transproceed.2009.01.013	Brandacher et al.	The Innsbruck Hand Transplant Program: Update at 8 Years First Transplant After the	2009	P1–P3: Multiple AR episodes, e.g., P1 at 55d, P2 at 9d, P3 at 51d, 60d, 601d	Banff I and II	N/A	P1: steroids; P2: steroid/ATG-resistant, basiliximab, alemtuzumab ×2, transient tacrolimus increase; P3: alemtuzumab for 3rd AR
DOI: 10.1016/j.transproceed.2009.01.020	Selvaggi et. al.	Abdominal Wall Transplantation: Surgical and Immunologic Aspect	2009	2 graft losses at POD 1, 6; 4 AR episodes treated	N/A	N/A	Steroid boluses, weaning protocol
DOI: 10.1016/j.transproceed.2009.01.018	Schneeberger et. al.	Alemtuzumab: Key for Minimization of Maintenance Immunosuppression in Reconstructive Transplantation?	2009	P1: AR POD 51; P2: AR POM 18; P3: AR POD 120, POD 221; P4: POD 30, POD 170	Banff I to III	Diffuse rash on palms/joints, hand swelling/rash, macules on hands/forearms	P1: methylprednisolone 500 mg (3d); 2nd AR: steroids + tacrolimus/clobetasol (ineffective), alemtuzumab (20 mg); P2: topical TAC/clobetasol, tacrolimus increased to 12 ng/mL, MMF 1 g BID, resolved in 1 month; P3: 3 methylprednisolone pulses for both AR; P4: lesions spontaneous resolution (POD 135), 2nd AR: methylprednisolone 1 g ×3 every other day
DOI: 10.1097/PRS.0b013e3181c15c4c	Siemionow et. al.	First U.S. Near-Total Human Face Transplantation: A Paradigm Shift for Massive Complex Injuries	2010	Rejection was reported	Banff III to IV	N/A	Solu-Medrol 1 g bolus, remission within 72h
DOI: 10.1097/SLA.0b013e318226a607	Barett et al.	Full Face Transplant: The First Case Report	2011	POD 3, POD 7, POD 15, POD 28, POD 75, POM 3	Banff I to III	Severe edema and hyperemia	3d: protocol unchanged; 28d: 3 bolus prednisone (1 g) → taper (250→60 mg/d); 75d: bolus 1 g + 0.5 g; 3m: thymoglobulin (1.5 g/kg), MMF → sirolimus (3 ng/mL)
DOI: 10.1111/j.1600-6143.2011.03503.x	Cavadas et al.	Bilateral Trans-humeral Arm Transplantation: Result at 2 years	2011	POM 6, POM 13, POM 26	Banff III	Intraepithelial T-cell migration	Methylprednisolone i.v. bolus
DOI: 10.1111/j.1600-6143.2010.03406.x	Lantieri et al.	Feasibility, Reproducibility, Risks and Benefits of Face Transplantation: A Prospective Study of Outcomes	2011	POD 0, POD 5, POD 28, POD 64	Banff I	N/A	Methylprednisolone i.v. pulses (3d), ATG if steroid-resistant
DOI: 10.1111/j.1600-6143.2010.03368.x	Pomahac et. al.	Restoration of Facial Form and Function After Severe Disfigurement from Burn Injury by a Composite Facial Allograft	2011	POD 17, 74, 107	Banff I and II	Facial redness	1st AR: methylpred 500 mg ×3; oral prednisolone (15–30 mg/d); topical clobetazol (d27–35, 37–45), tacrolimus (d107–113)
DOI: 10.1097/TP.0b013e31826c3915	Pei et. al.	A Report of 15 Hand Allotransplantations in 12 Patients and Their Outcomes in China	2012	P1, P5–P7: Rejection every year post-surgery; P2: 15 months; P3: once; P4: once; P8: at 6 months and 2 years; P9: at 1, 3, 5, and 6 years; P10: 7 months; P11: 4 weeks, 8 weeks, and 2 years; P12: 2 years	N/A	Pain, ischemic skin necrosis, swelling, rash, hand swelling, dorsal erythema, thumb papule	P1: steroids (1 g/d ×3), reduced to 10 mg/d; P2: amputation after unhealed rejection; P3: amputation due to infection; P4: increased steroids; P5–7: steroids (1 g/d ×3); P8: 2m AR resolved with steroids; P9–10: steroids (1 g/d ×3); P11: methylprednisolone/ATG (4 weeks), 8 weeks, 2y necrosis due to rejection; P12: steroids helped initially, necrosis at 2y
DOI: 10.1111/ajt.12715	Chandraker et al.	The Management of Antibody-Mediated Rejection in the First Presensitized Recipient of a Full-Face Allotransplant	2014	POD 12, 15, 19	Banff I and II	N/A	TPE, eculizumab (POD20, 22, 27), bortezomib
DOI: 10.1016/j.transproceed.2014.08.028	Kaminska et al.	Significant Infections After Hand Transplantation in a Polish Population	2014	Number of biopsy proven rejections: P1: 1, P2: 2, P3: 2, P4: 7, P5: 2	N/A	N/A	Corticosteroids, topical tacrolimus
DOI: 10.1111/ajt.13103	Diaz-Siso et al.	Initial Experience of Dual Maintenance Immunosuppression With Steroid Withdrawal in Vascular Composite Tissue Allotransplantation	2015	P1: POM34, POM56; P2: POM22; P3: POD20, POM17, POM34; P4: POD54, POM17, POM30; P5: POM16, POM26	Banff I to III	Redness, rosacea, facial erythema and edema, swelling, pain	P1: increased TAC/MMF, topical treatments; P2: TAC/MMF/dexamethasone, topical treatments, ATG ×2; P3: TAC/MMF/steroid bolus (SB)/taper (ST); P4: TAC/MMF/SB/ST; P5: same
DOI: 10.1155/2015/356459	Kanitakis et al.	Premalignant and Malignant Skin Lesions in Two Recipients of Vascularized Composite Tissue Allografts (Face, Hands)	2015	P1: none, P2: several episodes	N/A	Violaceous, scaly papules on dorsum of hands/fingers	Steroids i.v., ATG, oral steroids, alemtuzumab; SRL
DOI: 10.1371/journal.pone.0136235	Kim et al.	Clonal CD8+ T Cell Persistence and Variable Gene Usage Bias in a Human Transplanted Hand	2015	POD 717 rejection due to medication nonadherence	Banff I to III	Clinical signs of inflammation	Severe AR: 3 Solu-Medrol boluses → ATG → amputation at day 771
DOI: 10.1097/SAP.0000000000000758	Kuo et al.	The First Hand Allotransplantation in Taiwan A Report at 9 Months	2015	POM 3.5	Banff I and II	Mild erythema, biopsy showed dense perivascular mononuclear infiltrate	Topical clobetasol (0.05%), tacrolimus 0.1%
DOI: 10.1097/TP.0000000000000765	Petruzzo et. al.	Clinicopathological Findings of Chronic Rejection in a Face Grafted Patient	2015	1st AR: POD 41; 2nd AR: POD 103; 3rd AR: POD 186; 4th AR: POD 239; 5th AR: POD 474; 6th AR: POD 527; 7th AR: POD 540; 8th AR: POD 931	Banff I to III	Facial/oral mucosa edema, erythema, skin/mucosa biopsies showed basal vacuolization, CD3+/CD4+ T-cells, lichenoid changes, later skin sclerosis, dermal thickening	1st AR: 3 bolus steroids (15 mg/kg); 2nd: oral steroids (10 mg/kg ×10d); 3rd: 3 bolus steroids (15 mg/kg); 4th: oral steroids (10 mg/kg ×10d); 5th: 3 bolus steroids (850 mg); 6–7th: Campath-1 (20 mg); 8th: i.v. steroids
DOI: 10.1097/SLA.0000000000000627	Petruzzo et. al.	Outcomes After Bilateral Hand Allotransplantation A Risk/Benefit Ratio Analysis	2015	P1: POD 53 and POD 72; P2: POD 57, POD 86, POD 2759; P3: POD 16, POD 271, POD 635, POD 951, POD 1365, POD 1855; P4: POD 65; P5: POD 10, POD 350, POD 560	Banff II to III	Erythematous macules, lichenoid micropapules; biopsies: CD3+/CD4+ infiltrate, basal vacuolization, thrombosis	P1–5: increased steroids; P3 also ATG, Campath-1H; photochemotherapy 3m
DOI: 10.1097/PRS.0000000000002605	Aycart et al.	A Retrospective Analysis of Secondary Revisions after Face Transplantation: Assessment of Outcomes, Safety, and Feasibility	2016	Antibody-mediated rejection	Banff II	N/A	Steroid pulse, prednisone taper
DOI: 10.1097/PRS.0000000000002153	Selber et. al.	Simultaneous Scalp, Skull, Kidney, and Pancreas Transplant from a Single Donor	2016	POW 11	Banff II	Perivascular lymphocytes, CD3+ staining	Solu-Medrol i.v.
DOI: 10.1111/ajt.14440	Grahammer et al.	Benefits and limitations of belatacept in 4 hand-transplanted patients	2017	P1: 1 AR, P2: 7 AR, P3: 6 AR, P4: 4 AR	Banff I and II	Edema, numbness, tingling/burning, mild perivascular infiltrates	P1–P4: steroids, rituximab/ATG
DOI: 10.4103/ijps.IJPS_96_17	Iyer et al.	First two bilateral hand transplantations in India (Part 4): Immediate post-operative care, immunosuppression protocol and monitoring	2017	P1: POW2, POW4, POM4, POM8, POM9; P2: POM 1	Banff I to III	Lesions, color changes, unexplained swelling	P1: rituximab 2×500 mg; P2–P3: methylpred 500 mg ×3; P4: steroid taper
DOI: 10.1002/micr.30272	Özkan et al.	Face allotransplantation for various types of facial disfigurements: a series of five cases.	2017	P1: 12 AR from POY 1; P2: 1 at POY 1; P3: 1 at POM 15; P4: multiple after IS reduction; P5: 1 at POM 24	Banff I and II	Erythema, edema	P1: resolved with steroid pulses and tacrolimus increase; P2, P3, P5: resolved with topical tacrolimus, tacrolimus dose increase, and steroids
DOI: 10.1111/ajt.14910	Cendales et al.	*De novo* belatacept in clinical vascularized composite allotransplantation	2018	POM 8	N/A	Round erythematous macules, edematous papules	Rejection resolved with methylprednisolone 500 mg i.v. × 3d, rapid taper to prednisone 10 mg/d
DOI: 10.1097/SLA.0000000000002241	Cetrulo et al.	Penis Transplantation First US Experience	2018	POD 28, POD 32	Banff I to III	N/A	POD28: 2 days i.v. methylprednisolone; POD32: 3 days i.v. methylprednisolone 500 mg with taper and 4 days ATG (1.5 mg/kg/d)
DOI: 10.1080/23320885.2018.1431047	Fallahian et al.	Eponychial lesions following bilateral upper extremity vascular composite allotransplantation: a case report	2018	AR episode	Banff II	Minor rash	Oral prednisone and tacrolimus increased transiently → clinical and histological improvement
DOI: 10.4097/kjae.2018.71.1.66	Kwon et al.	Anesthetic management of the first forearm transplantation in Korea	2018	POD 6, POD 47	Banff I	Erythematous changes	Steroid pulse therapy and ATG as per immunosuppression protocol; topical tacrolimus applied
DOI: 10.1111/tri.13096	Weissenbacher et. al.	*De novo* donor-specific HLA antibodies after combined intestinal and vascularized composite allotransplantation — a retrospective study	2018	38.9% cases experienced AR	N/A	T-cell rejection in all; in 38.9% visible skin rejection	High-dose i.v. steroids (500 mg bolus ×3d); Alemtuzumab for steroid-resistant rejection
DOI: 10.1097/GOX.0000000000002995	Atia et al.	Synchronous Abdominal Wall and Small-bowel Transplantation: A 1-year Follow-up	2020	1 AR episode	Banff III	Rash or skin changes	W-VCA rejection: High-dose steroids (5 days), Thymoglobulin, Clobetasol gel (1 event)
DOI: 10.1097/PRS.0000000000007890	Govshievich et al.	Face Transplant: Current Update and First Canadian Experience	2020	AR episode	Banff I	No clinical signs	Methylprednisolone pulses i.v., increased oral prednisone, tacrolimus adjustment. Later: basiliximab and/or Solumedrol (no prednisone change)
DOI: 10.1111/tri.13752	Hautz et al.	Long-term outcome after hand and forearm transplantation – a retrospective study	2020	All patients experienced AR; P4: chronic rejection at POY 7, leading to amputation	Banff I to IV	Vasculitis-related vascular changes, skin lesions, tingling/burning	Steroid bolus and tacrolimus increase; resistant AR: thymoglobulin/alemtuzumab; rituximab for ABMR
DOI: 10.1097/TP.0000000000003241	Roy et. al.	Lymphocytic Vasculitis Associated With Mild Rejection in a Vascularized Composite Allograft Recipient: A Clinicopathological Study	2020	POD 50 and POD 56, POD 70, POD 138, POD 286	Banff I	Lymphocytic vasculitis (biopsy); lymphocytes in vessel walls, edema, endothelial swelling	Solumedrol 250 mg i.v. ×3, prednisone 0.15→0.5 mg/kg
DOI: 10.1016/j.trim.2021.101377	Azoury et al.	Successful transatlantic bilateral hand transplant in a young female highly sensitized to HLA class II antigens	2021	AR episode	Banff I and II	N/A	Betamethasone dipropionate 0.05% cream BID
DOI: 10.1055/a-2059-5570	Lee et al.	One Year Experience of the Hand Allotransplantation First Performed after Korea Organ Transplantation Act (21) Amendment	2023	POD 33, POD 41	Banff I to III	Diffuse swelling and erythema	1st rejection: 500 mg methylprednisolone ×3d → taper to 60 mg/d; 2nd: same; topical steroids/tacrolimus; MMF stopped 27d for neutropenia risk
DOI: 10.1016/j.ajt.2023.01.016	Murakami et al.	Low-dose interleukin-2 promotes immune regulation in face transplantation: A pilot study	2023	4 AR episodes (POM 2, 17, 30, 47)	Banff II and III	N/A	4 AR (Banff II–III at 2, 17, 30, 47 months); after 54m, tacrolimus, sirolimus (6–8 ng/mL), IL-2; Banff 2/3 AR, methylprednisolone pulse
DOI: 10.3389/frtra.2024.1339898	Zaccardelli et. al.	Case Report: Post-transplant lymphoproliferative disorder as a serious complication of vascularized composite allotransplantation	2024	POM 26, POM 37	Banff II and III	N/A	1st AR: topical tacrolimus, increased oral MMF/tacrolimus; 2nd: topical tacrolimus/clobetasol, oral tacrolimus increased

POD, Postoperative Day; POW, Postoperative Week; POM, Postoperative Month; POY, Postoperative Year; AR, Acute Rejection; ATG, Antithymocyte Globulin; BID, Twice Daily; i.v., Intravenous; p.o., Oral; MPS, Methylprednisolone; MMF, Mycophenolate Mofetil; SB, Steroid Bolus; ST, Steroid Taper; CMV, Cytomegalovirus; Tac, Tacrolimus; SRL, Sirolimus; TPE, Therapeutic Plasma Exchange; IVIG, Intravenous Immunoglobulin; ABMR, Antibody-Mediated Rejection; W-VCA, Whole-Vascularized Composite Allotransplant; Solu-Medrol ,Methylprednisolone; IS, Immunosuppression; NK, Natural Killer Cells; MAC, Macrolide Antibiotics; FOXP3, Forkhead Box P3; IL-2, Interleukin-2; Treg, Regulatory T Cells; CD, Cluster of Differentiation; PDS, Prednisone; i.m., Intramuscular; d, day; 3m, 3 months; 3d, 3 days; P1–P5, Patient 1 to Patient 5; AR episode, Rejection episode; T-cell, T lymphocytes; SB+ST, Steroid Bolus and Taper.

### 3.4 Success rates of acute rejection treatment

Success of rejection treatment was defined as preservation of the graft, even if the immunosuppressive treatment regimen was changed during that rejection episode. Unsuccessful treatment was, in turn, defined as graft loss. Out of 136 VCA cases, rejection treatment was reported as successful in n = 91 (67%) cases, while graft loss was reported in n = 12 (8.8%) cases.

Rejection treatments reported in face VCAs had a success rate of 100% with treatment durations of 3 days to 8 weeks. In all (n = 41; 30%) but n = 1 (0.7%) rejection, STR was used. Here, n = 5 (3.7%) cases received STR as single treatment, n = 4 (2.9%) in combination with ATG, or in combination with ATG, TAC, topical TAC and MMF in n = 3 (2.2%) cases.

In upper-extremity VCAs, n = 54 (40%) cases were reported as successful, while n = 9 (6.6%) were unsuccessful and resulted in graft loss. Of these, n = 4 (2.9%) discontinued immunosuppressive therapy owing to infection and VCA-unrelated surgical interventions. In all other cases (n = 45; 33%), STR, topical TAC and ATG were used. STR single therapy was the most frequent (n = 22; 16%) followed by STR combined with topical TAC (n = 10; 7.4%) or STR combined with ATG (n = 5; 3.7%). Treatment duration ranged from 2 days to 3 months.

In abdominal-wall transplants, all (n = 33; 24%) rejections were treated either via STR single therapy, STR + alemtuzumab, or STR + ATG. Treatment duration ranged from 3 to 5 days and no graft losses were reported.

At last, rejection in scalp VCA (n = 1; 0.7%) was successfully treated with STR therapy, whereas rejection in penile VCA (n = 1; 0.7%) was treated by STR and ATG dual therapy over 3 days. Full insights on acute rejection treatment are provided in **Table 3**.

### 3.5 Chronic rejections

Despite more than two decades of clinical experience in VCA, a universally accepted definition or staging system for chronic rejection (CR) is still lacking. Consensus efforts remain focused on acute, skin-predominant changes, leaving late fibrotic and vasculopathic lesions insufficiently characterized (22, 23). The few systematically documented cases illustrated that CR is most likely under-recognized rather than rare. At present, no validated treatment algorithm exists.

A clinical descriptive series of CR from Krezdorn et al., reviewed longitudinal protocol biopsies from seven face-transplant recipients (24). Three patients developed progressive, clinically subtle changes - premature ageing, telangiectasia along suture lines, tightening of the skin - that correlated with distinctive histology: epidermal thinning, follicular plugging, papillary-dermal sclerosis and a shift of type-I collagen towards the superficial dermis. Gene-expression profiling pointed to AP-1-pathway activation (c-Fos/JunB) as a putative driver of fibrosis. Notably, microvascular intimal hyperplasia was absent, underscoring that cutaneous CR might evolve independently.

Current therapeutic evidence after chronic rejection is constrained to two cases of facial retransplantation (15, 20). Both patients lost their first VCA graft due to chronic rejection. One patient developed Grade 2/3 Banff rejection on day 14, while the other presented with Grade III chronic antibody-mediated rejection involving erythema and mucosal tissues.

After retransplantation, acute rejection occurred and was successfully managed with methylprednisolone bolus therapy, supplemented by eculizumab in the first patient and alemtuzumab in the second patient due to refractory mucosal involvement.

In sum, the available evidence portrays chronic rejection in VCA as a heterogeneous, slowly evolving entity that is clinically subtle, histologically diverse and, to date, largely untreatable except by retransplantation.

## 4 Discussion

Acute and chronic rejection remain the central challenges to the long-term success of VCA. Despite surgical and medical advancements, these forms of rejection continue to limit broader clinical adoption and underline the need for optimized immunosuppressive strategies and targeted therapies (1, 25–27).

In our study, acute rejection was common, with most patients experiencing multiple episodes that were generally well-managed using corticosteroids, tacrolimus, and adjunct therapies, resulting in high success rates and relatively low rates of graft loss. In contrast, chronic rejection was rarely reported, poorly characterized, and remains a largely untreatable challenge in VCA, underscoring the need for further research to improve long-term outcomes.

Focusing on acute rejection, our results were in line with current literature. STR-based therapies remained the frontline strategy for acute rejection episodes in VCA, as confirmed by Alhefzi et al., who reported resolution in up to 70–80% of cases across different graft types, while also noting that inadequately treated acute rejection could contribute to chronic graft failure (1). Beyond STR, adjunct agents such as ATG, MMF, and TAC have been employed in cases of STR-resistant rejection or as combined therapy to intensify immunosuppression. Fischer et al. confirmed that acute rejection episodes were generally STR-responsive, with treatment success in over 85% of cases following timely intervention. The authors highlighted the importance of optimized triple immunosuppressive therapy to prevent recurrence (6). Hautz et al. described that acute rejection was often treated not only with systemic STR but also with adjunctive topical agents such as topical TAC, which allowed localized immunosuppression directly at the graft site while reducing the risks associated with systemic drug exposure (28). This was further confirmed by recent studies, demonstrating that the vast majority —over 80%— of VCA rejection episodes in hand and face transplants were successfully controlled with high-dose STR and immunosuppressive adjustments (e.g. alemtuzumab, donor bone marrow), and patient specific considerations such as human leukocyte antigen (HLA) matching (29). Interestingly, experimental approaches, such as localized tacrolimus delivery via intra-graft injection or hydrogel-eluting platforms, have shown promise in in extending graft survival up to 200 days in animal models while avoiding systemic side effects (30, 31). Fisher et al. evaluated emerging biologic and cell-based therapies in VCA, including regulatory T cell–based tolerance strategies, and proposed these approaches as promising avenues to enhance long-term graft survival while potentially reducing or even eliminating the need for lifelong systemic immunosuppression (32). At last, Etra et al. discussed the emerging use of targeted therapies, including antibody-based agents and costimulatory blockade, particularly in sensitized or complex VCA recipients, though these approaches remained largely experimental (33). Despite the overall success of corticosteroid-based therapies in treating acute rejection in VCA, approximately 20–30% of episodes do not respond adequately to standard immunosuppression. This observation suggested the involvement of additional, possibly unexplored, alloimmune pathways that contribute to treatment-resistant rejection. This underscored the need for further research to elucidate these underlying immunologic mechanisms and to develop more targeted, individualized treatment strategies (1, 25, 26, 34, 35).

In contrast to acute rejection, chronic allograft deterioration in VCA lacks a standardized consensus definition, which remains a critical barrier to effective management. Our review highlights that chronic rejection is characterized in the literature by subtle, insidious evolution—manifesting as late vasculopathy (myointimal hyperplasia) and tissue fibrosis (sclerosis, adnexal atrophy)—yet there is currently no unified diagnostic algorithm or grading system comparable to the Banff criteria for acute rejection (22). This definitional ambiguity directly impacts clinical practice: we found no established therapeutic protocols for chronic rejection. While early acute rejection is successfully managed with standardized pulse corticosteroids and topical immunosuppression, treatment for chronic rejection is highly heterogeneous and largely empirical, often relying on salvage therapies (e.g., plasmapheresis, lymphoid depletion) with inconsistent success (9, 36). Chronic rejection thus appears to represent irreversible graft injury resulting from cumulative or inadequately controlled immune responses. Future studies should focus on the development of standardized diagnostic criteria and the establishment of evidence-based treatment protocols, rather than relying on *ad hoc* management of graft failure (29, 37, 38). Ultimately, this might also improve or facilitate finding appropriate VCA donors (39).

In summary, our findings support a pragmatic, stepwise clinical protocol for VCA rejection management that can be tailored to the severity and biology of rejection in individual recipients. However, given the descriptive nature of the available literature and variability in reporting and treatment strategies, these observations should be interpreted cautiously and cannot be taken as establishing a definitive, universally applicable protocol.

High success rates of corticosteroid-based treatment in acute rejection episodes likely reflect the importance of prompt recognition and early intervention, which are key to preserving graft function. However, despite these successes, approximately one-fifth of acute rejection episodes did not respond adequately to standard immunosuppression, suggesting the existence of additional, as-yet unexplored, alloimmune pathways. This underscores the need for further research to better understand these complex mechanisms and develop more targeted, individualized therapies to improve long-term outcomes. For steroid-resistant episodes, our data support combination immunosuppressive strategies involving agents such as ATG, MMF, and tacrolimus, which have shown efficacy in intensifying treatment. Because of the anatomic accessibility of VCA grafts, clinical practice should readily incorporate topical immunosuppression like tacrolimus as an adjunct for skin-predominant rejection to minimize systemic toxicity. Furthermore, escalation to B-cell targeted therapies (e.g. Rituximab), plasmapheresis, IVIG or proteasome inhibitors (e.g. Bortezomib) should be considered, particularly in complex or antibody-mediated rejections. Finally, for VCA patients, medication adherence and close communication with transplant teams are critical to ensuring timely detection and management of rejection, as salvage therapies have shown very limited efficacy in chronic graft rejection. Individuals at higher immunologic risk or with a prior history of rejection should be particularly diligent in attending follow-up appointments and maintaining ongoing dialogue with their care providers, as early therapeutic adjustments can significantly improve long-term outcomes. Ultimately, effective rejection management is essential to safeguarding the long-term success of VCA and ensuring optimal outcomes of VCA surgery over time.

## 5 Limitations

Despite the comprehensive approach of this systematic review, several limitations must be acknowledged. First, the heterogeneity of study designs, patient cohorts, and treatment protocols limited the feasibility of a quantitative meta-analysis. To address this, we employed a structured narrative synthesis and strictly categorized interventions to identify consistent clinical patterns across diverse centers and surgeries, thereby providing a consolidated overview of rejection management strategies in this rare field. Many included studies were case reports or small case series, reducing generalizability and statistical robustness. However, given the prevalence of VCA, these reports constitute the entirety of the available evidence base, and by aggregating these data, our study offers one of the largest cumulative datasets currently available. Since a number of studies grouped hand, wrist, and more proximal reconstructions indiscriminately, these procedures were pooled under the umbrella term “upper-extremity VCA,” which may mask anatomical differences. We tried to mitigate this by focusing our analysis on systemic immunological outcomes rather than functional metrics, as rejection mechanisms are largely independent of the specific level of amputation. Reporting of chronic rejection was highly inconsistent regarding surveillance biopsies and histological terminology. We addressed this by applying a standardized definition of ‘treatment success’ (graft salvage vs. loss) across all studies, ensuring a clinically relevant endpoint that remains valid despite histological variability. However, the heterogeneity in our dataset still reinforces the critical need for evidence-based guidelines to standardize both the diagnosis and therapeutic management of chronic rejection in VCA. In this context, establishing a multinational, multicenter outcomes database with harmonized definitions and reporting standards would be crucial to facilitate knowledge transfer and enable better treatment and outcome comparability across VCA centers. Additionally, the reliance on retrospective data introduces potential publication bias favoring positive outcomes. We attempted to minimize this by conducting a comprehensive search strategy, which included reports of graft failure and explicitly discussing complications, providing a more balanced view of therapeutic risks. While the exclusion of non-English publications may have omitted some data, our search strategy covered all major international VCA centers, ensuring that the most clinically relevant cases were captured. Furthermore, key immunological variables such as HLA mismatches, donor-specific antibodies (DSA), and panel reactive antibody (PRA) levels were reported too inconsistently to permit meaningful extraction or comparison, and this lack of standardized immunologic data represents an additional limitation of the available literature. Similarly, the inconsistent and often non–episode-specific reporting of rejection symptoms, together with the lack of standardized data on the timing of initial treatment response and subsequent therapy escalation, prevented meaningful correlation of clinical manifestations and treatment kinetics with early versus late rejection, representing an additional limitation of the current evidence base. Moreover, because many studies reported immunosuppressive regimens incompletely or with insufficient detail, only the most commonly used agents could be meaningfully synthesized, limiting the inclusion of experimental or less frequently used therapies and underscoring the need for more structured and standardized reporting in future VCA research. Additionally, because QoL and psychosocial outcomes were reported only sparingly and without standardized tools, we explicitly note that future research should systematically evaluate QoL impacts to provide a more holistic understanding of long-term patient outcomes. Importantly, this gap extends beyond patient-reported measures like the effects of rejection episodes to broader psychosocial dimensions—such as public reception and acceptance—that are relevant for long-term implementation (40). Finally, meaningful statistical comparison was not feasible due to substantial heterogeneity in study design, reporting standards, outcome definitions, follow-up duration, and immunosuppressive regimens, and this limitation highlights the urgent need for more standardized, comprehensive, and longitudinal data to enable the type of robust analyses required to advance evidence-based rejection management.

## 6 Conclusion

This systematic review demonstrates that while acute rejection in VCA is frequent, it is often responsive to a standardized, stepwise protocol of pulse corticosteroids and topical adjuncts, although the available evidence is largely retrospective and heterogeneous and therefore does not yet allow firm comparative conclusions on the relative effectiveness of different strategies. Chronic rejection remains a critical and underexplored barrier, likely underdiagnosed due to the absence of standardized diagnostic criteria and consequently limited therapeutic options once it is established. Consequently, long-term graft survival currently relies on the prevention of chronic deterioration through early rejection management and rigorous surveillance rather than rescue. Future efforts should prioritize standardizing diagnostic definitions and developing targeted therapies to bridge this gap, ultimately supporting the broader and safer adoption of VCA.

## Data Availability

The original contributions presented in the study are included in the article/**Supplementary Material**. Further inquiries can be directed to the corresponding authors.
