# Supplementary material for: A systematic review of treatment strategies to combat acute and chronic rejection episodes in vascularized composite allotransplantation
Source: Front Immunol. 2026 Feb 2;17:1733221. doi: 10.3389/fimmu.2026.1733221 (PMC12907174; doi:10.3389/fimmu.2026.1733221)
Supplement: Supplementary file 1 [file Table1.docx]

**12.Supplementary digital content**

**Supplementary digital content 1.** Assessment of study quality using the Level of Evidence scoring system

| **DOI** | **Author** | **Title** | **Study type** | **Sample size** | **Level of Evidence** |
| --- | --- | --- | --- | --- | --- |
| DOI: 10.1016/s0140-6736(99)02062-0 | Dubernard et al. | Human hand allograft: re p o rt on first 6 months | CR | 1 | 4 |
| DOI: 10.1097/01.SLA.0000078945.70869.82 | Dubernard et al. | Functional Results of the First Human Double-Hand Transplantation | CR | 1 | 4 |
| doi:10.1016/j.jhsa.2004.05.007 | Gabl et al. | Bilateral Hand Transplantation: Bone Healing Under Immunosuppression with Tacrolimus, Mycophenolate Mofetil, and Prednisolone | CR | 1 | 4 |
| https://dx.doi.org/10.1097/01.tp.0000168454.68139.0a | Schneeberger et. al. | Cytomegalovirus-Related Complications in Human Hand Transplantation | CS | 18 | 4 |
| DOI: 10.1016/S0140- 6736(06)68935-6 | Devauchelle et al. | First human face allograft: early report | CR | 1 | 4 |
| https://dx.doi.org/10.1111/j.1600-6143.2006.01266.x | Schneeberger et. al. | Status 5 Years after Bilateral Hand Transplantation | CR | 1 | 4 |
| DOI: 10.1056/NEJMoa072828 | Dubernard et al. | Outcomes 18 Months after the First Human Partial Face Transplantation | CR | 1 | 4 |
| doi:10.1016/j.jhsa.2008.02.015 | Breidenbach et al. | Outcomes of the First 2 American Hand Transplants at 8 and 6 Years Posttransplant | CS | 2 | 4 |
| doi:10.1016/j.main.2008.02.002 | Herzberg et al. | Clinical evaluation of two bilateral hand allotransplantations at six and three years follow-up | CS | 2 | 4 |
| https://dx.doi.org/10.1111/j.1600-6143.2007.02105.x | Schneeberger et. al. | Atypical Acute Rejection After Hand Transplantation | CS | 4 | 4 |
| https://dx.doi.org/10.1016/j.surg.2008.06.025 | Ravindra et. al. | Hand transplantation in the United States: Experience with 3 patients | CS | 3 | 4 |
| doi:10.1016/j.transproceed.2009.01.013 | Brandacher et al. | The Innsbruck Hand Transplant Program: Update at 8 Years First Transplant After the | CS | 3 | 4 |
| https://dx.doi.org/10.1016/j.transproceed.2009.01.020 | Selvaggi et. al. | Abdominal Wall Transplantation: Surgical and Immunologic Aspect | CS | 14 | 4 |
| https://dx.doi.org/10.1016/j.transproceed.2009.01.018 | Schneeberger et. al. | Alemtuzumab: Key for Minimization of Maintenance Immunosuppression in Reconstructive Transplantation? | CS | 4 | 4 |
| http://dx.doi.org/10.1097/PRS.0b013e3181c15c4c | Siemionow et. al. | First U.S. Near-Total Human Face Transplantation: A Paradigm Shift for Massive Complex Injuries | CR | 1 | 4 |
| DOI: 10.1097/SLA.0b013e318226a607 | Barett et al. | Full Face Transplant: The First Case Report | CR | 1 | 4 |
| doi: 10.1111/j.1600-6143.2011.03503.x | Cavadas et al. | Bilateral Trans-humeral Arm Transplantation: Result at 2 years | CR | 1 | 4 |
| doi: 10.1111/j.1600-6143.2010.03406.x | Lantieri et al. | Feasibility, Reproducibility, Risks and Benefits of Face Transplantation: A Prospective Study of Outcomes | CR | 4 | 4 |
| https://dx.doi.org/10.1111/j.1600-6143.2010.03368.x | Pomahac et. al. | Restoration of Facial Form and Function After Severe Disfigurement from Burn Injury by a Composite Facial Allograft | CR | 1 | 4 |
| https://dx.doi.org/10.1097/TP.0b013e31826c3915 | Pei et. al. | A Report of 15 Hand Allotransplantations in 12 Patients and Their Outcomes in China | Cohort study | 12 | 3 |
| doi: 10.1111/ajt.12715 | Chandraker et al. | The Management of Antibody-Mediated Rejection in the First Presensitized Recipient of a Full-Face Allotransplant | CR | 1 | 4 |
| http://dx.doi.org/10.1016/j.transproceed.2014.08.028 | Kaminska et al. | Significant Infections After Hand Transplantation in a Polish Population | CS | 5 | 4 |
| doi: 10.1111/ajt.13103 | Diaz-Siso et al. | Initial Experience of Dual Maintenance Immunosuppression With Steroid Withdrawal in Vascular Composite Tissue Allotransplantation | CS | 5 | 4 |
| http://dx.doi.org/10.1155/2015/356459 | Kanitakis et al. | Premalignant and Malignant Skin Lesions in Two Recipients of Vascularized Composite Tissue Allografts (Face, Hands) | CS | 2 | 4 |
| DOI:10.1371/journal.pone.0136235 | Kim et al. | Clonal CD8+ T Cell Persistence and Variable Gene Usage Bias in a Human Transplanted Hand | CR | 1 | 4 |
| DOI: 10.1097/SAP.0000000000000758 | Kuo et al. | The First Hand Allotransplantation in Taiwan A Report at 9 Months | CR | 1 | 4 |
| https://dx.doi.org/10.1097/TP.0000000000000765 | Petruzzo et. al. | Clinicopathological Findings of Chronic Rejection in a Face Grafted Patient | CR | 1 | 4 |
| https://dx.doi.org/10.1097/SLA.0000000000000627 | Petruzzo et. al. | Outcomes After Bilateral Hand Allotransplantation A Risk/Benefit Ratio Analysis | CS | 5 | 4 |
| DOI: 10.1097/PRS.0000000000002605 | Aycart et al. | A Retrospective Analysis of Secondary Revisions after Face Transplantation: Assessment of Outcomes, Safety, and Feasibility | CS | 7 | 4 |
| https://dx.doi.org/10.1097/PRS.0000000000002153 | Selber et. al. | Simultaneous Scalp, Skull, Kidney, and Pancreas Transplant from a Single Donor | CR | 1 | 4 |
| DOI: 10.1111/ajt.14440 | Grahammer et al. | Benefits and limitations of belatacept in 4 hand-transplanted patients | CS | 4 | 4 |
| DOI: 10.4103/ijps.IJPS_96_17 | Iyer et al. | First two bilateral hand transplantations in India (Part 4): Immediate post‑operative care, immunosuppression protocol and monitoring | CS | 2 | 4 |
| DOI: 10.1002/micr.30272 | Özkan et al. | Face allotransplantation for various types of facial disfigurements: a series of five cases. | CS | 5 | 4 |
| DOI: 10.1111/ajt.14910 | Cendales et al. | De novo belatacept in clinical vascularized composite allotransplantation | CR | 1 | 4 |
| DOI: 10.1097/SLA.0000000000002241 | Cetrulo et al. | Penis Transplantation First US Experience | CR | 1 | 4 |
| https://doi.org/10.1080/23320885.2018.1431047 | Fallahian et al. | Eponychial lesions following bilateral upper extremity vascular composite allotransplantation: a case report | CR | 1 | 4 |
| https://doi.org/10.4097/kjae.2018.71.1.66 | Kwon et al. | Anesthetic management of the first forearm transplantation in Korea | CR | 1 | 4 |
| https://dx.doi.org/10.1111/tri.13096 | Weissenbacher et. al. | De novo donor-specific HLA antibodies after combined intestinal and vascularized composite allotransplantation — a retrospective study | Cohort study | 18 | 3 |
| DOI: 10.1097/GOX.0000000000002995 | Atia et al. | Synchronous Abdominal Wall and Small-bowel Transplantation: A 1-year Follow-up | CR | 1 | 4 |
| DOI: 10.1097/PRS.0000000000007890 | Govshievich et al. | Face Transplant: Current Update and First Canadian Experience | CR | 1 | 4 |
| doi:10.1111/tri.13752 | Hautz et al. | Long-term outcome after hand and forearm transplantation – a retrospective study | CS | 5 | 4 |
| https://dx.doi.org/10.1097/TP.0000000000003241 | Roy et. al. | Lymphocytic Vasculitis Associated With Mild Rejection in a Vascularized Composite Allograft Recipient: A Clinicopathological Study | CR | 1 | 4 |
| https://doi.org/10.1016/j.trim.2021.101377 | Azoury et al. | Successful transatlantic bilateral hand transplant in a young female highly sensitized to HLA class II antigens | CR | 1 | 4 |
| DOI: 10.1055/a-2059-5570. | Lee et al. | One Year Experience of the Hand Allotransplantation First Performed after Korea Organ Transplantation Act [35] Amendment | CR | 1 | 4 |
| https://doi.org/10.1016/j.ajt.2023.01.016 | Murakami et al. | Low-dose interleukin-2 promotes immune regulation in face transplantation: A pilot study | CS | 2 | 4 |
| https://dx.doi.org/10.3389/frtra.2024.1339898 | Zaccardelli et. al. | Case Report: Post-transplant lymphoproliferative disorder as a serious complication of vascularized composite allotransplantation | CR | 1 | 4 |
| *Legend: CR, case report; CS, case series* | | | | | |

**Supplementary digital content 2.** Assessment of study quality using the Newcastle-Ottawa Scale

| **DOI** | **Author** | **Title** | **Study type** | **Sample size** | **Selection** | **Comparability** | **Exposure** | **NOS-Score** |
| --- | --- | --- | --- | --- | --- | --- | --- | --- |
| DOI: 10.1016/s0140-6736(99)02062-0 | Dubernard et al. | Human hand allograft: re p o rt on first 6 months | CR | 1 | 2 | 1 | 2 | 5 |
| DOI: 10.1097/01.SLA.0000078945.70869.82 | Dubernard et al. | Functional Results of the First Human Double-Hand Transplantation | CR | 1 | 2 | 1 | 2 | 5 |
| doi:10.1016/j.jhsa.2004.05.007 | Gabl et al. | Bilateral Hand Transplantation: Bone Healing Under Immunosuppression with Tacrolimus, Mycophenolate Mofetil, and Prednisolone | CR | 1 | 2 | 1 | 2 | 5 |
| https://dx.doi.org/10.1097/01.tp.0000168454.68139.0a | Schneeberger et. al. | Cytomegalovirus-Related Complications in Human Hand Transplantation | CS | 18 | 2 | 1 | 2 | 5 |
| DOI: 10.1016/S0140- 6736(06)68935-6 | Devauchelle et al. | First human face allograft: early report | CR | 1 | 2 | 1 | 2 | 5 |
| https://dx.doi.org/10.1111/j.1600-6143.2006.01266.x | Schneeberger et. al. | Status 5 Years after Bilateral Hand Transplantation | CR | 1 | 2 | 1 | 2 | 5 |
| DOI: 10.1056/NEJMoa072828 | Dubernard et al. | Outcomes 18 Months after the First Human Partial Face Transplantation | CR | 1 | 2 | 1 | 2 | 5 |
| doi:10.1016/j.jhsa.2008.02.015 | Breidenbach et al. | Outcomes of the First 2 American Hand Transplants at 8 and 6 Years Posttransplant | CS | 2 | 2 | 1 | 2 | 5 |
| doi:10.1016/j.main.2008.02.002 | Herzberg et al. | Clinical evaluation of two bilateral hand allotransplantations at six and three years follow-up | CS | 2 | 2 | 1 | 2 | 5 |
| https://dx.doi.org/10.1111/j.1600-6143.2007.02105.x | Schneeberger et. al. | Atypical Acute Rejection After Hand Transplantation | CS | 4 | 2 | 1 | 2 | 5 |
| https://dx.doi.org/10.1016/j.surg.2008.06.025 | Ravindra et. al. | Hand transplantation in the United States: Experience with 3 patients | CS | 3 | 2 | 1 | 2 | 5 |
| doi:10.1016/j.transproceed.2009.01.013 | Brandacher et al. | The Innsbruck Hand Transplant Program: Update at 8 Years First Transplant After the | CS | 3 | 2 | 1 | 2 | 5 |
| https://dx.doi.org/10.1016/j.transproceed.2009.01.020 | Selvaggi et. al. | Abdominal Wall Transplantation: Surgical and Immunologic Aspect | CS | 14 | 2 | 1 | 2 | 5 |
| https://dx.doi.org/10.1016/j.transproceed.2009.01.018 | Schneeberger et. al. | Alemtuzumab: Key for Minimization of Maintenance Immunosuppression in Reconstructive Transplantation? | CS | 4 | 2 | 1 | 2 | 5 |
| http://dx.doi.org/10.1097/PRS.0b013e3181c15c4c | Siemionow et. al. | First U.S. Near-Total Human Face Transplantation: A Paradigm Shift for Massive Complex Injuries | CR | 1 | 2 | 1 | 2 | 5 |
| DOI: 10.1097/SLA.0b013e318226a607 | Barett et al. | Full Face Transplant: The First Case Report | CR | 1 | 2 | 1 | 2 | 5 |
| doi: 10.1111/j.1600-6143.2011.03503.x | Cavadas et al. | Bilateral Trans-humeral Arm Transplantation: Result at 2 years | CR | 1 | 2 | 1 | 2 | 5 |
| doi: 10.1111/j.1600-6143.2010.03406.x | Lantieri et al. | Feasibility, Reproducibility, Risks and Benefits of Face Transplantation: A Prospective Study of Outcomes | CR | 4 | 2 | 1 | 2 | 5 |
| https://dx.doi.org/10.1111/j.1600-6143.2010.03368.x | Pomahac et. al. | Restoration of Facial Form and Function After Severe Disfigurement from Burn Injury by a Composite Facial Allograft | CR | 1 | 2 | 1 | 2 | 5 |
| https://dx.doi.org/10.1097/TP.0b013e31826c3915 | Pei et. al. | A Report of 15 Hand Allotransplantations in 12 Patients and Their Outcomes in China | Cohort study | 12 | 2 | 2 | 2 | 6 |
| doi: 10.1111/ajt.12715 | Chandraker et al. | The Management of Antibody-Mediated Rejection in the First Presensitized Recipient of a Full-Face Allotransplant | CR | 1 | 2 | 1 | 2 | 5 |
| http://dx.doi.org/10.1016/j.transproceed.2014.08.028 | Kaminska et al. | Significant Infections After Hand Transplantation in a Polish Population | CS | 5 | 2 | 1 | 2 | 5 |
| doi: 10.1111/ajt.13103 | Diaz-Siso et al. | Initial Experience of Dual Maintenance Immunosuppression With Steroid Withdrawal in Vascular Composite Tissue Allotransplantation | CS | 5 | 2 | 1 | 2 | 5 |
| http://dx.doi.org/10.1155/2015/356459 | Kanitakis et al. | Premalignant and Malignant Skin Lesions in Two Recipients of Vascularized Composite Tissue Allografts (Face, Hands) | CS | 2 | 2 | 1 | 2 | 5 |
| DOI:10.1371/journal.pone.0136235 | Kim et al. | Clonal CD8+ T Cell Persistence and Variable Gene Usage Bias in a Human Transplanted Hand | CR | 1 | 2 | 1 | 2 | 5 |
| DOI: 10.1097/SAP.0000000000000758 | Kuo et al. | The First Hand Allotransplantation in Taiwan A Report at 9 Months | CR | 1 | 2 | 1 | 2 | 5 |
| https://dx.doi.org/10.1097/TP.0000000000000765 | Petruzzo et. al. | Clinicopathological Findings of Chronic Rejection in a Face Grafted Patient | CR | 1 | 2 | 1 | 2 | 5 |
| https://dx.doi.org/10.1097/SLA.0000000000000627 | Petruzzo et. al. | Outcomes After Bilateral Hand Allotransplantation A Risk/Benefit Ratio Analysis | CS | 5 | 2 | 1 | 2 | 5 |
| DOI: 10.1097/PRS.0000000000002605 | Aycart et al. | A Retrospective Analysis of Secondary Revisions after Face Transplantation: Assessment of Outcomes, Safety, and Feasibility | CS | 7 | 2 | 1 | 2 | 5 |
| https://dx.doi.org/10.1097/PRS.0000000000002153 | Selber et. al. | Simultaneous Scalp, Skull, Kidney, and Pancreas Transplant from a Single Donor | CR | 1 | 2 | 1 | 2 | 5 |
| DOI: 10.1111/ajt.14440 | Grahammer et al. | Benefits and limitations of belatacept in 4 hand-transplanted patients | CS | 4 | 2 | 1 | 2 | 5 |
| DOI: 10.4103/ijps.IJPS_96_17 | Iyer et al. | First two bilateral hand transplantations in India (Part 4): Immediate post‑operative care, immunosuppression protocol and monitoring | CS | 2 | 2 | 1 | 2 | 5 |
| DOI: 10.1002/micr.30272 | Özkan et al. | Face allotransplantation for various types of facial disfigurements: a series of five cases. | CS | 5 | 2 | 1 | 2 | 5 |
| DOI: 10.1111/ajt.14910 | Cendales et al. | De novo belatacept in clinical vascularized composite allotransplantation | CR | 1 | 2 | 1 | 2 | 5 |
| DOI: 10.1097/SLA.0000000000002241 | Cetrulo et al. | Penis Transplantation First US Experience | CR | 1 | 2 | 1 | 2 | 5 |
| https://doi.org/10.1080/23320885.2018.1431047 | Fallahian et al. | Eponychial lesions following bilateral upper extremity vascular composite allotransplantation: a case report | CR | 1 | 2 | 1 | 2 | 5 |
| https://doi.org/10.4097/kjae.2018.71.1.66 | Kwon et al. | Anesthetic management of the first forearm transplantation in Korea | CR | 1 | 2 | 1 | 2 | 5 |
| https://dx.doi.org/10.1111/tri.13096 | Weissenbacher et. al. | De novo donor-specific HLA antibodies after combined intestinal and vascularized composite allotransplantation — a retrospective study | Cohort study | 18 | 2 | 2 | 2 | 6 |
| DOI: 10.1097/GOX.0000000000002995 | Atia et al. | Synchronous Abdominal Wall and Small-bowel Transplantation: A 1-year Follow-up | CR | 1 | 2 | 1 | 2 | 5 |
| DOI: 10.1097/PRS.0000000000007890 | Govshievich et al. | Face Transplant: Current Update and First Canadian Experience | CR | 1 | 2 | 1 | 2 | 5 |
| doi:10.1111/tri.13752 | Hautz et al. | Long-term outcome after hand and forearm transplantation – a retrospective study | CS | 5 | 2 | 1 | 2 | 5 |
| https://dx.doi.org/10.1097/TP.0000000000003241 | Roy et. al. | Lymphocytic Vasculitis Associated With Mild Rejection in a Vascularized Composite Allograft Recipient: A Clinicopathological Study | CR | 1 | 2 | 1 | 2 | 5 |
| https://doi.org/10.1016/j.trim.2021.101377 | Azoury et al. | Successful transatlantic bilateral hand transplant in a young female highly sensitized to HLA class II antigens | CR | 1 | 2 | 1 | 2 | 5 |
| DOI: 10.1055/a-2059-5570. | Lee et al. | One Year Experience of the Hand Allotransplantation First Performed after Korea Organ Transplantation Act [35] Amendment | CR | 1 | 2 | 1 | 2 | 5 |
| https://doi.org/10.1016/j.ajt.2023.01.016 | Murakami et al. | Low-dose interleukin-2 promotes immune regulation in face transplantation: A pilot study | CS | 2 | 2 | 1 | 2 | 5 |
| https://dx.doi.org/10.3389/frtra.2024.1339898 | Zaccardelli et. al. | Case Report: Post-transplant lymphoproliferative disorder as a serious complication of vascularized composite allotransplantation | CR | 1 | 2 | 1 | 2 | 5 |
| *Legend: CR, case report; CS, case series* | | | | | | | | |

**Supplemental digital content 3.** Search strategy for PubMed/MEDLINE, Web of Science, and EMBASE databases

| **Database** | **Search strategy** | **Results** |
| --- | --- | --- |
| PubMed/MEDLINE | ("VCA" OR "VCA transplantation" OR "vascularized composite allotransplantation" OR "vascularized composite allograft" OR "vascularized composite tissue transplantation" OR "vascularized allogenic tissue" OR "vascularized allograft" OR "composite tissue allotransplant" OR "composite tissue transplant" OR "composite tissue allograft")  AND  ("Graft Rejection"[MeSH] OR "Transplant Rejection"[MeSH] OR "graft rejection" OR "allograft rejection" OR "chronic rejection" OR "acute rejection" OR "immune rejection" OR "antibody-mediated rejection" OR "cell-mediated rejection" OR "rejection response" OR "rejection mechanism") | 952 |
| Web of Science | TS=("VCA" OR "VCA transplantation" OR "vascularized composite allotransplantation" OR "vascularized composite allograft" OR "vascularized composite tissue transplantation" OR "vascularized allogenic tissue" OR "vascularized allograft" OR "composite tissue allotransplant" OR "composite tissue transplant" OR "composite tissue allograft")  AND  TS=("graft rejection" OR "transplant rejection" OR "allograft rejection" OR "chronic rejection" OR "acute rejection" OR "immune rejection" OR "antibody-mediated rejection" OR "cell-mediated rejection" OR "rejection response" OR "rejection mechanism") | 371 |
| EMBASE | ('vascularized composite allotransplantation'/exp OR 'vascularized composite allotransplantation' OR 'VCA' OR 'vascularized composite allograft' OR 'vascularized composite tissue transplantation' OR 'vascularized allogenic tissue' OR 'vascularized allograft' OR 'composite tissue allotransplant' OR 'composite tissue transplant' OR 'composite tissue allograft')  AND  ('graft rejection'/exp OR 'graft rejection' OR 'transplant rejection' OR 'allograft rejection' OR 'chronic rejection' OR 'acute rejection' OR 'immune rejection' OR 'antibody-mediated rejection' OR 'cell-mediated rejection' OR 'rejection response' OR 'rejection mechanism') | 1014 |
